# Systemic inflammation and health outcomes in patients receiving treatment for atherosclerotic cardiovascular disease

**DOI:** 10.1093/eurheartj/ehae557

**Published:** 2024-08-30

**Authors:** Faizan Mazhar, Anne-Laure Faucon, Edouard L Fu, Karolina E Szummer, Jimmi Mathisen, Sofia Gerward, Simon Bertram Reuter, Nikolaus Marx, Roxana Mehran, Juan-Jesus Carrero

**Affiliations:** Department of Medical Epidemiology and Biostatistics, Campus Solna, Karolinska Institutet, Nobels väg 12A, 171 65 Stockholm, Sweden; Department of Medical Epidemiology and Biostatistics, Campus Solna, Karolinska Institutet, Nobels väg 12A, 171 65 Stockholm, Sweden; Department of Medical Epidemiology and Biostatistics, Campus Solna, Karolinska Institutet, Nobels väg 12A, 171 65 Stockholm, Sweden; Department of Clinical Epidemiology, Leiden University Medical Center, Leiden, The Netherlands; Department of Cardiology, Karolinska Institutet, Karolinska University Hospital, Huddinge, Stockholm, Sweden; Novo Nordisk A/S, Søborg, Denmark; Novo Nordisk A/S, Søborg, Denmark; Novo Nordisk A/S, Søborg, Denmark; Department of Internal Medicine I, RWTH Aachen University, Aachen, Germany; Mount Sinai School of Medicine, Mount Sinai Health System, New York City, NY, USA; Department of Medical Epidemiology and Biostatistics, Campus Solna, Karolinska Institutet, Nobels väg 12A, 171 65 Stockholm, Sweden; Division of Nephrology, Department of Clinical Sciences, Danderyd Hospital, Danderyd, Sweden

**Keywords:** Inflammation, CRP, SCREAM, Cardiovascular disease, Prognosis, Prediction, Biomarker

## Abstract

**Background and Aims:**

The burden and outcomes of inflammation in patients with atherosclerotic cardiovascular disease (ASCVD) are not well defined beyond the controlled settings of trials and research cohorts.

**Methods:**

This was an observational study of ASCVD adults undergoing C-reactive protein testing in Stockholm’s healthcare (2007–21). After excluding C-reactive protein tests associated with acute illness or medications/conditions that bias C-reactive protein interpretation, systemic inflammation was evaluated over a 3-month ascertainment window. Determinants of C-reactive protein ≥ 2 mg/L were explored with logistic regression. C-reactive protein categories were compared via negative-binomial/Cox regression for subsequent healthcare resource utilization and occurrence of major adverse cardiovascular events, heart failure hospitalization, and death.

**Results:**

A total of 84 399 ASCVD adults were included (46% female, mean age 71 years, 59% with C-reactive protein ≥ 2 mg/L). Female sex, older age, lower kidney function, albuminuria, diabetes, hypertension, and recent anaemia were associated with higher odds of C-reactive protein ≥ 2 mg/L. The use of renin–angiotensin system inhibitors, antiplatelets, and lipid-lowering therapy was associated with lower odds. Over a median of 6.4 years, compared with C-reactive protein < 2 mg/L, patients with C-reactive protein ≥ 2 mg/L had higher rates of hospitalizations, days spent in hospital, outpatient consultations, and dispensed medications (*P* < .05 for all). They also had a higher rate of major adverse cardiovascular events [hazard ratio (HR) 1.30; 95% confidence interval (CI) 1.27–1.33], heart failure (HR 1.24; 95% CI 1.20–1.30), and death (HR 1.35; 95% CI 1.31–1.39). Results were consistent across subgroups and granular C-reactive protein categories and robust to the exclusion of extreme C-reactive protein values or early events.

**Conclusions:**

Three in five adults with ASCVD have systemic inflammation, which is associated with excess healthcare resource utilization and increased rates of cardiovascular events and death.

## Structured Graphical Abstract

**Structured Graphical Abstract:**
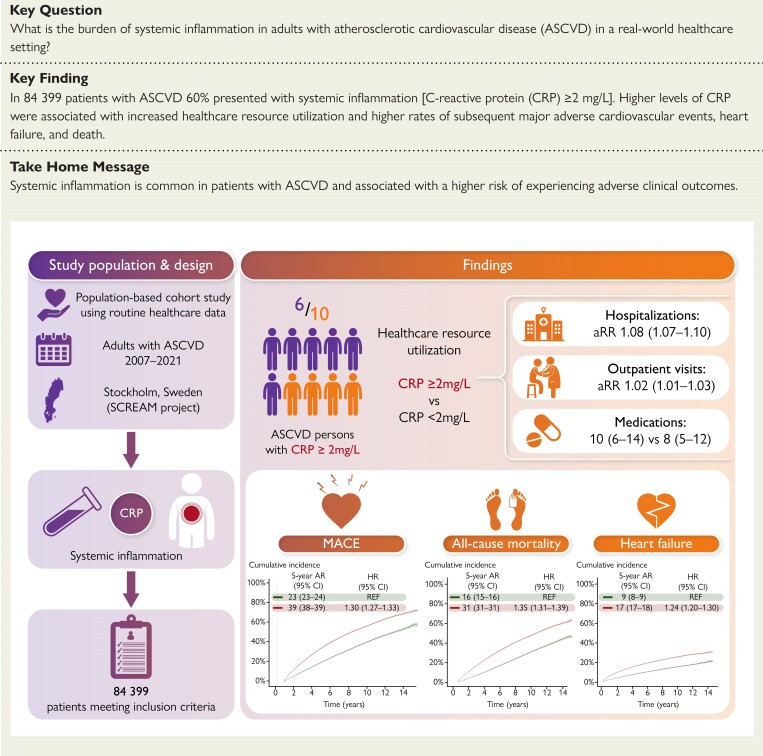
Systemic inflammation and health outcomes in adults receiving treatment for atherosclerotic cardiovascular disease. AR, absolute risk; aRR, adjusted rate ratio; ASCVD, atherosclerotic cardiovascular disease; CI, confidence interval; CRP, C-reactive protein; HCRU, healthcare resource utilization; HR, hazard ratio; MACE, major adverse cardiovascular events; SCREAM, Stockholm CREAtinine Measurements.

**See the editorial comment for this article ‘Universal screening for hsCRP in patients with atherosclerotic disease: a Major therapeutic opportunity’, by G. Liuzzo and P. M Ridker, https://doi.org/10.1093/eurheartj/ehae565.**

## Introduction

Atherosclerotic cardiovascular disease (ASCVD) remains the leading cause of morbidity and mortality worldwide.^1^ Despite the routine use of lipid-lowering, blood pressure-lowering, and antithrombotic therapy, people with ASCVD still face a significant risk of recurrent cardiovascular events, with 30% experiencing a major adverse cardiovascular event (MACE) over a decade.^2^ Besides traditional risk factors, systemic low-grade inflammation is nowadays recognized as a driving force of atherosclerotic disease progression and plaque destabilization. Systemic low-grade inflammation may also explain, in part, the unaddressed residual risk of atherothrombotic events of patients with ASCVD.^3–6^ Targeted anti-inflammatory pharmacological treatments *de facto* lower the rates of cardiovascular events in patients with myocardial infarction or chronic coronary disease.^7–10^

C-reactive protein is a well-established plasma marker of inflammation widely used in clinical practice.^11^ Recent meta-analyses of observational studies have shown that C-reactive protein can predict cardiovascular events in the general population or populations free from cardiovascular disease.^12–15^ There are considerably fewer studies evaluating the prevalence of systemic low-grade inflammation in populations with ASCVD or what C-reactive protein elevations in these patients signify in terms of health outcomes.^16–18^ Available studies come primarily from research cohorts^19–30^ and clinical trials,^31–35^ being often limited by relatively small sample sizes, short follow-up, or focusing on one subtype of ASCVD. In addition, the controlled settings of trials and research cohorts, subjected to strict inclusion/exclusion criteria and monitoring protocols, may not reflect the heterogeneous general population seeking healthcare.

Given the paucity of evidence regarding inflammation in ASCVD, this study provides real-world evidence on the distribution of C-reactive protein, its clinical determinants, and associated adverse health outcomes. Such evidence may guide future trials and inform clinical decisions on the usefulness of screening/monitoring inflammation in these patients as well as on the size and target populations who may benefit from inflammation-targeted therapies.

## Methods

### Data source

This study uses data from the Stockholm CREAtinine Measurements (SCREAM) project,^36^ a complete healthcare utilization cohort from the region of Stockholm, Sweden. A single healthcare provider in the Stockholm region provides universal and tax-funded healthcare to 20%–25% of the population of Sweden. Using unique personal identification numbers, SCREAM linked regional and national administrative databases that hold complete information on demographics, healthcare utilization, laboratory tests undertaken, dispensed drugs, diagnoses, and vital status without loss of follow-up. At present, SCREAM has data from January 2006 to December 2021. The regional ethical review board in Stockholm approved the study; informed consent was not deemed necessary because all data were de-identified at the Swedish Board of Health and Welfare.

### Study design and study population

The study population included all adult (>18 years old) patients with a first/incident clinical diagnosis of ASCVD during 1 January 2007 to 31 September 2021. Atherosclerotic cardiovascular disease was defined as receiving a diagnosis of coronary, cerebrovascular, or peripheral artery disease (see Supplementary data online, *Table S1*). The date of the first available diagnosis during the study period that qualified for ASCVD constituted the cohort entry date. All subsequent measurements of C-reactive protein performed in any source of care (in either primary care, outpatient specialist, or inpatient care) were then extracted.

Real-world healthcare differs from controlled settings, with C-reactive protein testing being done by indication and judgment of the attending physician. Given the multiple indications of C-reactive protein tests, several patient- and test-specific exclusion criteria were applied to avoid, as much as possible, healthcare use-related biases. To minimize the confounding introduced by conditions influencing systemic C-reactive protein levels, we excluded C-reactive protein tests occurring within the 30 days after the ASCVD diagnosis, C-reactive protein tests taken during an inpatient stay or emergency department visit, and abnormally elevated C-reactive protein tests (>20 mg/L), presumably all of them indicative of acute illness. We also excluded C-reactive protein tests followed by the prescription of antibiotics, antivirals, or antimycotics within 7 days on the assumption that infection was the reason for C-reactive protein testing (see Supplementary data online, *Table S2*). Likewise, we excluded C-reactive protein levels during the following 3 months of these infections as they may relate to the monitoring and/or resolution of the infection event. The remaining C-reactive protein tests were considered eligible for our study and assumed to likely reflect systemic inflammation.

We then selected the first eligible C-reactive protein level per patient to define a 3-month baseline window. This was done to minimize the potential for reverse causation bias (i.e. a C-reactive protein test is part of the workout for diagnosis of disease that leads to death). Thus, patients were required to survive at least 3 months after the first eligible C-reactive protein measurement, and the geometric mean of all C-reactive protein tests available during these 3 months was calculated as a reflection of their systemic inflammation. Finally, we excluded patients with comorbidities and/or long-term medications that modify or bias the interpretation of C-reactive protein concentrations (chronic infections—hepatitis, tuberculosis, or HIV—and ongoing use of corticosteroids or immunosuppressive drugs). The end of this 3-month period was considered the index date of the study, where study covariates were derived, and follow-up for clinical outcomes began (see Supplementary data online, *Figure S1*). To clarify, the latest date that an eligible participant could be included in the analysis was 1 September 2021, and the participant needed to survive 3 months with study baseline on 31 December 2021.

### Study exposure

The study exposure was C-reactive protein, taken as a proxy of systemic inflammation. In Stockholm region, three central laboratories provide services to the region, and their methods are regularly audited by the Government agency EQUALIS (www.equalis.se/en) to ensure standardization, reproducibility, and consistency across the region’s unified healthcare. Over the years, Stockholm laboratories have used different assays or analysers to measure C-reactive protein. In all of them, C-reactive protein levels were measured in plasma samples, by either immunochemistry or turbidimetry methods. We discarded three methods that were non-sensitive with a detection limit of 3 mg/L and that accounted for about 5% of C-reactive protein tests. We considered the remaining eight assays, which accounted for 95% of the C-reactive protein tests available and were all high-sensitivity C-reactive protein assays with a detection limit of 1 mg/L or lower.

Given the absence of a universally accepted definition for systemic low-grade inflammation in ASCVD, our primary exposure considered two categories of C-reactive protein (<2 vs. ≥2 mg/L), in alignment with the criterion used in clinical trials and definitions of residual inflammatory risk.^7^ Our secondary exposure considered four categories of C-reactive protein (≤1, 1–3, 3–10, and >10 to ≤20 mg/L), consistent with the thresholds that define normality in the general population and allowing us to explore more granular dose–response associations.

### Study covariates

Study covariates were derived at the index date and included age, sex, comorbid conditions, ongoing medications, and laboratory tests. Supplementary data online, *Table S3* provides a detailed description of covariate definitions. Medications were considered concomitant if dispensed within 6 months before the index date. Dispensations were obtained from the National Prescribed Drug Register, which has complete coverage of all prescribed medications dispensed in Swedish pharmacies. Laboratory values considered outpatient measurements of serum/plasma creatinine, haemoglobin, total and low-density lipoprotein cholesterol, and urinary albumin (albuminuria). If these tests were not available at index date, we selected the closest in time with a look-back window of 12 months. Missing values (i.e. the laboratory test was not ordered during the defined window) were coded as a missing category. Glomerular filtration rate was estimated (eGFR) using the chronic kidney disease epidemiology collaboration equation^37^ and categorized into three levels of severity (>60, 30–60, and <30 mL/min/1.73 m²) consistent with the KDIGO classification.^38^ Patients undergoing maintenance dialysis or kidney transplantation were identified via linkage with the Swedish Renal Registry and included in the lowest eGFR category. Albuminuria tests considered both urinary albumin to creatinine ratio (UACR, either on-spot urine sample, morning, evening, or 24-h collection) and dipstick albuminuria, and their concentrations were categorized (<30, 30–300, and >300 mg/g) according to the KDIGO classification.^38^

### Study outcomes

We evaluated healthcare resource utilization metrics associated with baseline C-reactive protein, including the annual rate of hospitalizations and outpatient specialist visits, the length of hospital stay, mean number of days spent in the hospital, and number of unique medications dispensed.

Clinical study outcomes included MACE (a composite of hospitalization for myocardial infarction, stroke, or all-cause death), hospitalization for heart failure, and death (all-cause and cardiovascular and non-cardiovascular death). Algorithms to define these events are detailed in Supplementary data online, *Table S4* and were ascertained by linkage with Stockholm’s health system database and the Swedish death registry, which records vital status and reported causes of death for all Swedish citizens. Patients were followed from the index date until the occurrence of an event, emigration, or the end of follow-up (31 December 2021), whichever occurred first. To clarify, the hypothetical last-included participant with baseline on 31 December 2021 would be censored on the same day and do not contribute with follow-up to the analysis of outcomes.

### Statistical analyses

Descriptive statistics are shown as median with first and third quartile (Q1–Q3), mean with standard deviation, or frequencies with corresponding percentages. Logistic regression analysis was employed to identify baseline conditions associated with systemic inflammation. Negative binomial regression was used to model the relative rate of hospitalizations and outpatient specialist consultations within each C-reactive protein category. The models accounted for differential follow-up lengths by incorporating an offset term. The count variable in our models was the number of hospitalizations or outpatient specialist visits per year. These models were adjusted for a set of a priori selected baseline covariates.

The cumulative incidence of each event was estimated by the competing risk method, in which death from other causes was considered a competing risk. Comparisons between exposure groups used Gray's test. Multivariable-adjusted cause-specific Cox proportional hazards regression was used to estimate hazard ratios (HRs) for the association between C-reactive protein and clinical study outcomes. The proportional hazard assumption was checked using log(−log[S]) plots and Schoenfeld residuals against time. Non-linear associations between baseline C-reactive protein levels and study outcomes were explored using restricted cubic splines, with a smooth function applied to the logarithm of C-reactive protein due to its skewed distribution.

Subgroup analyses tested the consistency of our findings across strata of age, sex, eGFR, UACR, and LDL cholesterol categories, presence of diabetes comorbidity, myocardial infarction, heart failure, recent cancer (3 years), and use of lipid-lowering therapy. We evaluated the presence of effect heterogeneity through multiplicative interaction terms.

Sensitivity analyses considered (i) redefining our baseline C-reactive protein level with the minimum C-reactive protein level encountered per patient during the 3-month eligibility window (instead of the geometric mean); (ii) excluding patients with C-reactive protein > 10 mg/L at baseline; and (iii) excluding events occurring within the first 6 or 12 months of follow-up to evaluate the impact of potential reverse causation bias (i.e. suspicion of disease was the reason for C-reactive protein testing).

## Results

### Study cohort selection

**Figure 1:**
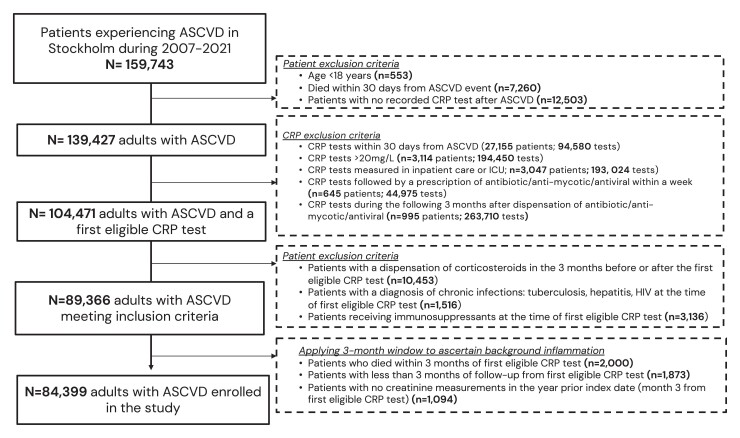
Patient selection flow chart. ASCVD, atherosclerotic cardiovascular disease

Following inclusion and exclusion criteria, a total of 84 399 adults with ASCVD were included (*Figure 1*). During the 3-month eligibility window that defined baseline systemic inflammation, there were 111 344 C-reactive protein tests [median (Q1–Q3) (min; max) 1(1–1) (1; 40) tests per patient). Most participants (79%) had one C-reactive protein measurement during this window, and the remaining 21% had between 2 and 44 measurements (see Supplementary data online, *Figure S2*).

### Baseline characteristics and conditions associated with residual inflammation

At baseline, 59% of study participants had C-reactive protein levels ≥ 2 mg/L. Their mean age was 71 years, and 54% were men (*Table 1*). The most common ASCVD condition was stroke (43%), followed by myocardial infarction (37%) and angina (30%). In 85% of cases, the index C-reactive protein was measured more than 6 months after the ASCVD event. Supplementary data online, *Table S5* shows patient characteristics, according to more granular categories of C-reactive protein levels.

**Table 1:** Baseline characteristics of adults with atherosclerotic cardiovascular disease, overall and by C-reactive protein categories

Characteristic	Overall, *N* = 84 399	C-reactive protein level < 2 mg/L, *n* = 34 526 (41%)	C-reactive protein level ≥ 2–20 mg/L, *n* = 49 873 (59%)	SMD
**C-reactive protein** in mg/L; median [Q1–Q3]	2.0 [1.0, 5.4]	0.9 [0.9, 1.0]	4.9 [3.0, 9.0]	1.70
**Age** in years; mean [SD]	71 [13]	69 [13]	72 [13]	0.25
**Men**	45 840 (54%)	19 742 (57%)	26 098 (52%)	−0.09
**Time since ASCVD**				
<6 months	12 800 (15%)	5322 (15%)	7478 (15%)	−0.01
≥6 months to <2 years	41 407 (49%)	17 318 (50%)	24 089 (48%)	−0.03
2 to <5 years	20 861 (25%)	8138 (24%)	12 723 (26%)	0.04
≥5 years	9331 (11%)	3748 (11%)	5583 (11%)	0.01
**Haemoglobin** in g/dL (*n* = 79 410)	135 [16]	137 [15]	133 [17]	0.28
**LDL cholesterol** in mmol/L (*n* = 52 966)	2.52 [1.01]	2.42 [0.98]	2.60 [1.03]	0.17
**eGFR categories**				
≥60 mL/min/1.73 m^2^	66 380 (79%)	29 000 (84%)	37 380 (75%)	−0.18
≥30 to ≤59 mL/min/1.73 m^2^	16 158 (19%)	5111 (15%)	11 047 (22%)	0.19
≤30 mL/min/1.73 m^2^	1861 (2.2%)	415 (1.2%)	1446 (2.9%)	0.12
**Albuminuria categories**				
<30 mg/g	40 831 (48%)	17 899 (52%)	22 932 (46%)	−0.11
30–300 mg/g	10 643 (13%)	3739 (11%)	6904 (14%)	0.09
>300 mg/g	5087 (6.0%)	1593 (4.6%)	3494 (7.0%)	0.10
Missing	27 838 (33%)	11 295 (33%)	16 543 (33%)	0.00
**Comorbid conditions**				
Diabetes mellitus	18 735 (22%)	6496 (19%)	12 239 (25%)	0.14
Hypertension	58 246 (69%)	22 262 (64%)	35 984 (72%)	0.17
Myocardial infraction	31 442 (37%)	13 813 (40%)	17 629 (35%)	−0.10
CABG	3137 (3.7%)	1221 (3.5%)	1916 (3.8%)	−0.02
PCI	15 171 (18%)	7757 (22%)	7414 (15%)	0.01
Angina	24 998 (30%)	10 848 (31%)	14 150 (28%)	−0.07
Heart failure	14 646 (17%)	4298 (12%)	10 348 (21%)	0.22
Stroke/TIA	36 088 (43%)	14 218 (41%)	21 870 (44%)	0.05
Peripheral vascular disease	9427 (11%)	2936 (8.5%)	6491 (13%)	0.15
Atrial fibrillation	17 005 (20%)	5498 (16%)	11 507 (23%)	0.18
Rheumatoid disease	9776 (12%)	2997 (8.7%)	6779 (14%)	0.16
Chronic respiratory disease	15 356 (18%)	5197 (15%)	10 159 (20%)	0.14
Recent Cancer (3-years)	10 722 (13%)	3674 (11%)	7048 (14%)	0.11
Recent anaemia (1-year)	27 988 (33%)	9119 (26%)	18 869 (38%)	0.25
Dyslipidaemia	60 951 (72%)	27 069 (78%)	33 882 (68%)	−0.24
**Ongoing medications**				
Antiplatelets	54 998 (65%)	23 967 (69%)	31 031 (62%)	−0.15
ACEIs/ARBs	45 730 (54%)	18 739 (54%)	26 991 (54%)	−0.00
MRAs	4873 (5.8%)	1507 (4.4%)	3366 (6.7%)	0.10
β-Blockers	44 017 (52%)	17 808 (52%)	26 209 (53%)	0.02
SGLT-2 inhibitors	705 (0.8%)	297 (0.9%)	408 (0.8%)	−0.00
Diuretics	21 020 (25%)	6119 (18%)	14 901 (30%)	0.29
Calcium channel blockers	22 681 (27%)	8678 (25%)	14 003 (28%)	0.07
Statins/PCSK-9i, Ezetimibe (LLT)	49 809 (59%)	22 864 (66%)	26 945 (54%)	0.25
High intensity LLT	17 981 (36.1%)	8909 (40%)	9072 (33%)	−0.17
Moderate intensity LLT	29 435 (59.1%)	12 582 (56%)	16 853 (62%)	0.14
Low intensity LLT	2393 (4.8%)	995 (4%)	1398 (5%)	0.03
NSAIDs	10 328 (12%)	3831 (11%)	6497 (13%)	0.06

ACEIs, angiotensin-converting enzyme inhibitors; ARBs, angiotensin II receptor blockers; ASCVD, atherosclerotic cardiovascular disease; CABG, coronary artery bypass graft; eGFR, estimated glomerular filtration rate; LLT, lipid-lowering treatment; MRAs, mineralocorticoid receptor antagonists; NSAIDs, non-steroidal anti-inflammatory drugs; PCI, percutaneous coronary intervention; PCSK-9i, pro-protein convertase subtilisin/kexin type 9 inhibitors; SD, standard deviation; SGLT-2, sodium-glucose co-transporter-2; SMD, standardized mean difference; TIA, transient ischaemic attack.

The baseline conditions associated with C-reactive protein ≥ 2 mg/L in both univariable- and multivariable-adjusted analyses included female sex, older age, lower eGFR, higher levels of albuminuria, and comorbid conditions such as diabetes mellitus, hypertension, recent anaemia and cancer, and chronic respiratory and rheumatic diseases. Conversely, the use of renin–angiotensin system inhibitors, antiplatelets, and lipid-lowering therapy was associated with lower odds of elevated C-reactive protein (see Supplementary data online, *Table S6*).

### Healthcare resource utilization associated with C-reactive protein

Over a median follow-up of 6.4 (3.1–9.8) years, and compared with participants with C-reactive protein < 2 mg/L, participants with C-reactive protein ≥ 2 mg/L experienced a higher annual rate of hospitalizations (75 vs. 51 per 100 person-years), spent more days in hospital per year (mean 8 vs. 3.75 days), had a higher annual rate of outpatient specialist visits, and dispensed more medications (*Table 2*). More granular categories of C-reactive protein evidenced a clear dose–response association with all healthcare resource utilization metrics.

**Table 2:** Healthcare resource utilization associated with baseline C-reactive protein categories

	C-reactive protein < 2mg/L (*n* = 34 526)	C-reactive protein ≥ 2mg/L (*n* = 49 873)	C-reactive protein ≤ 1 mg/L (*n* = 19 072)	C-reactive protein > 1–3 mg/L (*n* = 31 450)	C-reactive protein > 3–10 mg/L (*n* = 25 770)	C-reactive protein > 10–20 mg/L (*n* = 8107)
**Subsequent hospitalizations**						
Median (Q1–Q3) no. of hospitalizations per year	0.18 (0.00–0.72)	0.41 (0.00–1.29)	0.22 (0.00–0.69)	0.22 (0.00–0.87)	0.42 (0.00–1.32)	0.68 (0.00–1.96)
Median (Q1–Q3) no. of days spent in hospital per year	0.26 (0.00–2.72)	1.1 (0.00–6.82)	0.34 (0.00–2.83)	0.34 (0.00–3.58)	1.16 (0.00–6.94)	2.83 (0.00–2.02)
Total no. of hospitalizations/total person-years	93 297/183 177	183 928/245 567	56 800/117 478	89 185/150 000	97 322/127 044	33 918/34 221
Incidence rate per 100 person-years	50.9 (50.7–51.2)	74.9 (74.7–75.1)	48.3 (48.1–48.6)	59.5 (59.2–59.7)	76.6 (76.4–76.8)	99.1 (99–99.2)
Crude incidence rate ratios	REF	1.26 (1.25–1.28)	REF	1.19 (1.17–1.21)	1.35 (1.33–1.37)	1.71 (1.68–1.75)
Adjusted incidence rate-ratios^a^	REF	1.08 (1.07–1.10)	REF	1.08 (1.07–1.10)	1.13 (1.11–1.15)	1.24 (1.22–1.27)
**Subsequent outpatient specialist consultations**						
Median (Q1–Q3) no. of visits	2.64 (1.12–5.34)	3.01 (1.24–6.25)	2.61 (1.17–5.10)	2.76 (1.14–5.70)	3.07 (1.26–6.39)	3.08 (1.17–6.94)
Total no. of visits/total person-years	829 053/183 177	1 436 880/245 567	500 388/117 478	750 044/150 000	773 451/127 044	242 050/34 221
Incidence rate per 100 person-years	452.6 (442.9–462.3)	585.1 (575.6–594.7)	425.9 (414.1–437.7)	500 (488.7–511.3)	608.8 (595.2–622.4)	707.3 (679.1–735.5)
Crude incidence rate ratios	REF	1.19 (1.18–1.20)	REF	1.44 (1.42–1.46)	1.46 (1.44–1.48)	1.81 (1.78–1.85)
Adjusted incidence rate ratios^a^	REF	1.02 (1.01–1.03)	REF	1.27 (1.25–1.28)	1.19 (1.18–1.21)	1.29 (1.27–1.31)
**Subsequent dispensed medications**						
Median (Q1–Q3) no. of unique medications dispensed per year	8 (5–12)	10 (6–14)	8 (5–12)	9 (6–13)	10 (6–14)	10 (7–15)

^a^Adjusted for age, sex, time since ASCVD, eGFR, albuminuria, comorbidities (diabetes mellitus, hypertension, chronic respiratory disease, cancer, MI, angina, heart failure, peripheral vascular disease, stroke/TIA, atrial fibrillation, and rheumatoid diseases), undertaken procedures (coronary artery bypass grafting and percutaneous coronary intervention), and ongoing medications [antiplatelet, NSAIDs, angiotensin-converting enzyme inhibitors/angiotensin receptor blockers, mineralocorticoid-receptor antagonists, β blocker, SGLT-2i, diuretics, calcium channel blockers, digoxin, lipid-lowering treatment (statins, PCSk9i, and ezetimibe)].

### Risk of adverse cardiovascular outcomes associated with C-reactive protein

**Figure 2:**
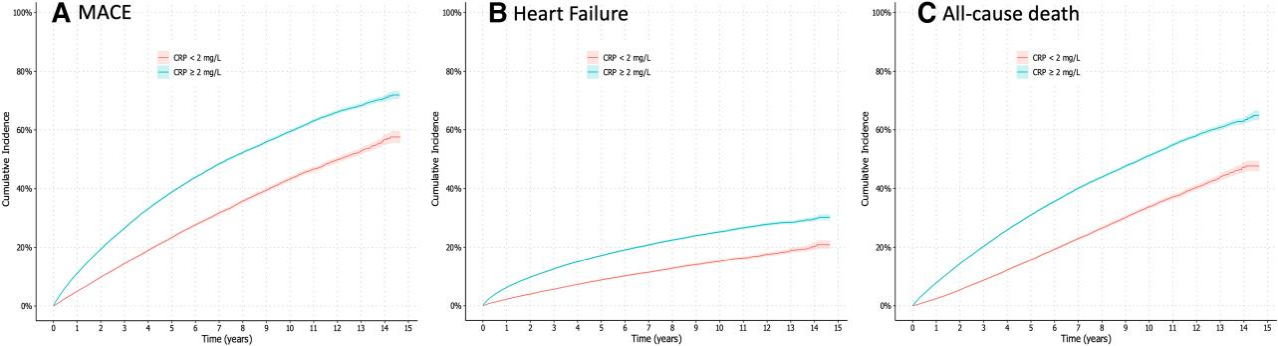
Cumulative incidence curves depicting the cumulative incidence of (*A*) major adverse cardiovascular events, (*B*) heart failure hospitalization, and (*C*) all-cause mortality associated with baseline C-reactive protein categories. MACE, major adverse cardiovascular events

During follow-up, the study observed 30 801 cases of MACE, 12 782 hospitalizations due to heart failure, and 24 954 deaths, of which 6772 were attributed to cardiovascular-related causes, and 18 155 to non-cardiovascular causes. The incidence rates and absolute risks for these outcomes were consistently higher in participants with elevated levels of C-reactive protein (*Figures 2* and see Supplementary data online, *Figure S3*). For example, the 5-year absolute risk of MACE for participants with C-reactive protein < 2 mg/L was 23% [95% confidence interval (CI) 23%–24%], considerably lower than the 39% (95% CI 38%–39%) absolute risk of participants with C-reactive protein ≥ 2 mg/L.

The adjusted relative rate of events in participants with C-reactive protein ≥ 2 mg/L (vs. C-reactive protein < 2 mg/L) was 30% higher for MACE (adjusted HR 1.30; 95% CI 1.27–1.33), 24% higher for heart failure (HR 1.24; 95% CI 1.20–1.30), and 35% higher for all-cause mortality (HR 1.35; 95% CI 1.31–1.39) (*Table 3*). When evaluating causes of death, the association between C-reactive protein ≥ 2 mg/L and cardiovascular mortality was similar in magnitude (HR 1.29; 95% CI 1.22–1.36) as for non-cardiovascular mortality (HR 1.24; 95% CI 1.20–1.28). A direct dose–response relationship was observed across more granular C-reactive protein categories. In restricted cubic spline curves, the rate of adverse events increased exponentially with higher C-reactive protein concentrations (see Supplementary data online, *Figure S4*), plateauing at a C-reactive protein value of ≈5–6 mg/L for the outcome of heart failure.

**Table 3:** Number of events, incidence rates, absolute risks and hazard ratios for adverse clinical outcomes associated with baseline C-reactive protein categories

	No. of events/No. of patients	Incidence rate per 1000 person-years (95% CI)	5-year absolute risks (95% CI)	Crude HR (95% CI)	Adj. HR (95% CI)^a^
**Major adverse cardiovascular events**					
C-reactive protein < 2 mg/L	9331/34 526	55 (53.9–56.1)	23% (23%, 24%)	REF.	REF.
C-reactive protein ≥ 2 mg/L	21 470/49 873	96.1 (94.8–97.3)	39% (38%, 39%)	1.74 (1.70–1.78)	1.30 (1.27–1.33)
C-reactive protein ≤ 1 mg/L	5659/19 072	52.0 (50.7–53.3)	22% (21%, 23%)	REF.	REF.
C-reactive protein > 1–3 mg/L	9220/31 450	66.8 (65.5–68.1)	28% (27%, 28%)	1.27 (1.23–1.32)	1.09 (1.06–1.13)
C-reactive protein > 3–10 mg/L	11 105/25 770	96.3 (94.6–98.0)	39% (39%, 40%)	1.84 (1.78–1.9)	1.34 (1.30–1.38)
C-reactive protein > 10–20 mg/L	4817/8107	155.7 (151.7–159.8)	55% (53%, 56%)	2.96 (2.85–3.07)	1.61 (1.54–1.67)
**Heart failure hospitalization**					
C-reactive protein < 2 mg/L	3409/34 526	17.3 (16.7–17.9)	9% (8%, 9%)	REF.	REF.
C-reactive protein ≥ 2 mg/L	9373/49 873	32.9 (32.2–33.6)	17% (17%, 18%)	1.91 (1.84–1.99)	1.24 (1.20–1.30)
C-reactive protein ≤ 1 mg/L	1961/19 072	15.6 (14.9–16.3)	8.1% (7.7%, 8.5%)	REF.	REF.
C-reactive protein > 1–3 mg/L	3792/31 450	23.3 (22.5–24.0)	12% (11%, 12%)	1.44 (1.37–1.52)	1.13 (1.07–1.20)
C-reactive protein > 3–10 mg/L	4892/25 770	33.2 (32.3–34.1)	17% (17%, 18%)	2.10 (1.99–2.21)	1.27 (1.20–1.34)
C-reactive protein > 10–20 mg/L	2137/8107	46.9 (45.0–48.9)	24% (23%, 25%)	2.96 (2.78–3.15)	1.31 (1.22–1.39)
**All-cause mortality**					
C-reactive protein < 2 mg/L	6912/34 526	37.8 (36.9–38.6)	16% (15%, 16%)	REF.	REF.
C-reactive protein ≥ 2 mg/L	18 042/49 873	73.5 (72.5–74.6)	31% (31%, 31%)	1.95 (1.90–2.00)	1.35 (1.31–1.39)
C-reactive protein ≤ 1 mg/L	4170/19 072	35.5 (34.5–36.6)	14% (14%, 15%)	REF.	REF.
C-reactive protein > 1–3 mg/L	7092/31 450	47.3 (46.2–48.4)	20% (19%, 20%)	1.34 (1.29–1.39)	1.10 (1.06–1.14)
C-reactive protein > 3–10 mg/L	9338/25 770	73.6 (72.1–75.0)	31% (31%, 32%)	2.08 (2.00–2.15)	1.39 (1.34–1.44)
C-reactive protein > 10–20 mg/L	4354/8107	127.3 (123.8–130.9)	48% (47%, 49%)	3.61 (3.46–3.77)	1.68 (1.61–1.75)
**Cardiovascular mortality**					
C-reactive protein < 2 mg/L	1736/34 526	8.5 (8.1–8.9)	4.1% (3.9%, 4.4%)	REF.	REF.
C-reactive protein ≥ 2 mg/L	5063/49 873	16.4 (15.9–16.8)	8.9% (8.6%, 9.2%)	1.95 (1.84–2.05)	1.29 (1.22–1.36)
C-reactive protein ≤ 1 mg/L	1002/19 072	7.6 (7.2–8.1)	3.7% (3.4%, 4.0%)		
C-reactive protein > 1–3 mg/L	1885/31 450	10.9 (10.4–11.4)	5.3% (5.0%, 5.6%)	1.41 (1.31–1.53)	1.16 (1.07–1.25)
C-reactive protein > 3–10 mg/L	2598/25 770	16.2 (15.6–16.8)	8.9% (8.5%, 9.3%)	2.12 (1.97–2.28)	1.36 (1.26–1.47)
C-reactive protein > 10–20 mg/L	1314/8107	26 (24.6–27.4)	15% (14%, 16%)	3.39 (3.12–3.68)	1.57 (1.44–1.71)
**Non-cardiovascular mortality**					
C-reactive protein < 2 mg/L	5176/34 526	27 (26.3–27.8)	12% (11%, 12%)	REF.	REF.
C-reactive protein ≥ 2 mg/L	12 979/49 873	47.5 (46.7–48.3)	22% (22%, 23%)	1.76 (1.7–1.81)	1.24 (1.20–1.28)
C-reactive protein ≤ 1 mg/L	3168/19 072	25.9 (25–26.8)	11% (10%, 11%)	REF.	REF.
C-reactive protein > 1–3 mg/L	5207/31 450	32.7 (31.8–33.5)	14% (14%, 15%)	1.26 (1.2–1.31)	1.04 (1.00–1.09)
C-reactive protein > 3–10 mg/L	6740/25 770	47.7 (46.6–48.8)	22% (22%, 23%)	1.84 (1.76–1.92)	1.25 (1.19–1.30)
C-reactive protein > 10–20 mg/L	3040/8107	72.4 (69.9–74.9)	33% (32%, 34%)	2.79 (2.65–2.93)	1.36 (1.29–1.43)

^a^Adjusted for age, sex, time since ASCVD, eGFR, albuminuria, comorbidities (diabetes mellitus, hypertension, chronic respiratory disease, cancer, MI, angina, heart failure, peripheral vascular disease, stroke/TIA, atrial fibrillation, and rheumatoid diseases), undertaken procedures (coronary artery bypass grafting and percutaneous coronary intervention), and ongoing medications [antiplatelet, NSAIDs, angiotensin-converting enzyme inhibitors/angiotensin receptor blockers, mineralocorticoid-receptor antagonists, β blocker, SGLT-2i, diuretics, calcium channel blockers, digoxin, lipid-lowering treatment (statins, PCSk9i, and ezetimibe)].

### Subgroup analyses

**Figure 3:**
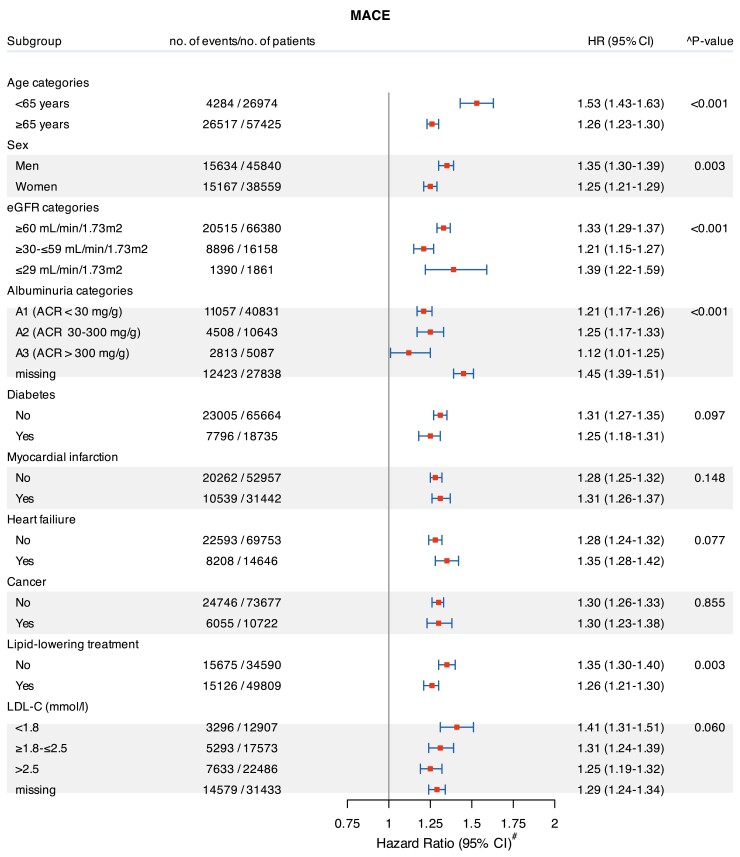
Subgroup analyses: forest plots of C-reactive protein ≥ 2 mg/L (vs. C-reactive protein < 2 mg/L) and rate of major adverse cardiovascular events. ^Models were adjusted (when appropriate) for age, sex, time since atherosclerotic cardiovascular disease, estimated glomerular filtration rate, albuminuria, comorbidities (diabetes mellitus, hypertension, chronic respiratory disease, cancer, myocardial infarction, angina, heart failure, peripheral vascular disease, stroke/transient ischaemic attack, atrial fibrillation, and rheumatoid diseases), undertaken procedures (coronary artery bypass grafting and percutaneous coronary intervention), and ongoing medications (antiplatelet, non-steroidal anti-inflammatory drugs, angiotensin-converting enzyme inhibitors/angiotensin receptor blockers, mineralocorticoid receptor antagonists, beta-blockers, sodium-glucose co-transporter 2 inhibitors, diuretics, calcium channel blockers, digoxin, lipid-lowering treatment (statins, pro-protein convertase subtilisin/kexin type 9 inhibitors, and ezetimibe). CI, confidence interval; eGFR, estimated glomerular filtration rate; HR, hazard ratio; MACE, major adverse cardiovascular events

The relationship between C-reactive protein and rate of study outcomes was consistent across strata of age, sex, eGFR, albuminuria, LDL cholesterol, comorbidities, and use of lipid-lowering therapy. Multiplicative interaction terms suggest that the magnitude of the association was stronger in some subgroups, but it was elevated regardless (*Figure 3*; see Supplementary data online, *Figures S4 and S5*).

### Sensitivity analyses

Re-defining baseline C-reactive protein with the minimum concentration observed during the 3-month eligibility window [median C-reactive protein_minimum_, 2 (1–5.4) mg/L] provided similar results (see Supplementary data online, *Table S7*). Associations with study outcomes remained after the exclusion of patients with C-reactive protein > 10 mg/L (see Supplementary data online, *Table S8*). The exclusion of events occurring during the first 6 or 12 months of follow-up minimally attenuated the magnitude of the estimates (see Supplementary data online, *Table S9*), suggesting reverse causation bias to be possibly low.

## Discussion

This large evaluation of adults with stable ASCVD managed in outpatient routine care found that a majority (59%) presented with systemic low-grade inflammation as indicated by a C-reactive protein ≥ 2 mg/L. Patients with C-reactive protein ≥ 2 mg/L had higher healthcare resource utilization and a higher rate of MACE, heart failure, and death. The associations were consistent across more granular categories of C-reactive protein and predefined subgroups and were robust to a range of sensitivity analyses (*Structured Graphical Abstract*).

Quantifying the prevalence of systemic inflammation in ASCVD is important for healthcare planning: 59% of patients in our study had C-reactive protein ≥ 2 mg/L, and 40% had C-reactive protein levels > 3 mg/L. Previous studies from clinical trials or prospective research cohort studies^10,12,13,30,34,33^ were limited by small sample sizes, the focus on one specific ASCVD condition or having inclusion/exclusion criteria that hinder generalizability. Because our analysis reflects populations accessing healthcare, it could be argued that factors or conditions influencing the decision for C-reactive protein testing might explain our results. Our careful design and exclusions of C-reactive protein tests potentially affected by acute illness and biasing conditions attempted to minimize this, and the external validity of our analysis may be found in a recent report of the US NHANES screening cohort,^39^ whereby 55% of ASCVD participants (*n* = 12.722) had C-reactive protein ≥ 2 mg/L. A study strength is that C-reactive protein levels were computed as the median of all C-reactive protein measurements within a 3-month period. However, a limitation is that most patients (79%) only had one C-reactive protein test taken in their healthcare records. This may have introduced some misclassification bias of the exposure (i.e. the C-reactive protein taken does not reflect the true systemic inflammation of the patient), but at the same time, it is the information available to clinicians to guide clinical decisions. In a research cohort of 9.005 patients with ASCVD from the Netherlands, 61% had C-reactive protein ≥ 2 mg/L at inclusion.^30^ Similar research cohorts of people with ASCVD from China describe a lower inflammation prevalence of 8%,^27^ in accordance with the reported lower C-reactive protein concentrations for predominantly Asian ethnicities.^15^

While routine C-reactive protein testing is not yet universally recommended by current European^40^ or US^41^ guidelines for secondary ASCVD prevention, our study shows that C-reactive protein is informative. The observed increased healthcare resource utilization across higher C-reactive protein levels is a novel finding, and one that translates into higher costs to society over and above the already excess cost of managing ASCVD.^42,43^ We also observed a direct association between systemic low-grade inflammation and subsequent rates of adverse health outcomes, with a magnitude and consistency not dissimilar from previous smaller ASCVD research cohorts,^30,27^ or studies of various cardiovascular disease conditions and settings.^44^ C-reactive protein production is part of the nonspecific acute-phase response to most forms of inflammation, infection, and tissue damage.^45^ This non-specificity may explain that C-reactive protein ≥ 2 mg/L in our study was similarly associated with both cardiovascular and non-cardiovascular related mortality. We acknowledge as study limitation that causes of death in routine care are not always confirmed by autopsies, so the possibility of misclassification exists. However, regardless of the pathway represented by systemic inflammation, our study supports the value of C-reactive protein assessment and integration of this information into the clinical decision process for risk stratification,^46^ investigation of underlying causes of inflammation, and possibly treatment intensification.^47,48^

As seen with LDL cholesterol,^49^ where ‘higher is worse’, our spline curve analyses show risks of C-reactive protein not to be confined to a simple binary classification, but rather show a linear association similar to that observed in primary cardiovascular prevention studies.^12^ This is relevant because in trials, participants most likely to achieve the target C-reactive protein concentration of <2 mg/L were, naturally, those who started out with lower baseline values. Those who started with higher baseline C-reactive protein values were less likely to reach the target and not benefit from therapy.^50^ Therefore, our study is also useful to understand the complex array of comorbid conditions contributing to residual inflammatory risk that may benefit from stricter monitoring, discussions conveying risk and encouraging lifestyle and/or treatment changes. Besides the expected conditions, like older age and select comorbidities, ongoing or recent anaemia was associated with higher odds of inflammation, in line with our current understanding of inflammation-induced suppression of erythropoiesis and impaired iron homoeostasis.^51^ Two markers of kidney damage, eGFR and albuminuria, also predicted the probability to present with elevated C-reactive protein, consistent with the described changes that kidney injury induces in innate and adaptive immunity.^52,53^ Inflammation was also lower among users of renin–angiotensin system inhibitors, attributed to the impact of renin–angiotensin system blockade on improving innate and adaptive immunity.^54^ Finally, participants using lipid-lowering therapy had lower C-reactive protein levels, in line with the demonstrated effects of this therapy in reducing inflammation.^34,55^ However, our study also showed that the observed risk of adverse health outcomes persists regardless of the use of these therapies.

The study has several strengths, including large sample size, rich data and complete coverage of a large region, and virtually no losses to follow-up. The setting of the Swedish universal tax-funded healthcare is also a strength as it provides observations not impacted by selection bias from disparate access to healthcare or disaggregated data sources. The study also has additional limitations. It represents the healthcare of a single region of predominantly Caucasian ethnicity during a defined period. Extrapolation to other regions, ethnic diversities, or periods should be done with caution. We lacked information on important covariates such as body mass index or smoking habits that may also impact C-reactive protein levels. Finally, intrinsic to all observational studies, we cannot infer causality in the associations observed. Although C-reactive protein may be a marker of inflammation rather than part of the causal pathway between inflammation and cardiovascular disease,^56^ randomized clinical trials have shown the efficacy of anti-inflammatory drugs in reducing the risk of cardiovascular events in secondary cardiovascular prevention.^7,8,9^

To conclude, by analysing C-reactive protein levels in adults with ASCVD accessing routine healthcare, this study illustrates the high prevalence of systemic inflammation and the strong association between even mild elevations in C-reactive protein levels and subsequent healthcare resource utilization as well as cardiovascular event recurrence. Expanding previous evidence from trials and research cohorts, this observational study therefore provides real-world evidence on the size of the population potentially benefited by targeted anti-inflammatory strategies for secondary ASCVD prevention and on the value of C-reactive protein testing in routine clinical for risk stratification and management.

## Supplementary data

Supplementary data are available at *European Heart Journal* online.

## Supplementary Material

ehae557_Supplementary_Data

## Data Availability

The data underlying this article cannot be shared publicly due to the privacy of individuals who participated in the study. The data may be shared on reasonable request for academic research collaborations that fulfil GDPR as well as national and institutional ethics regulations and standards by contacting Prof. Juan Jesus Carrero (juan.jesus.carrero@ki.se).

## Pre-registered Clinical Trial Number

None supplied.
