# Supplementary material for: Systemic inflammation and health outcomes in patients receiving treatment for atherosclerotic cardiovascular disease
Source: Eur Heart J. 2024 Aug 30;45(44):4719–30. doi: 10.1093/eurheartj/ehae557 (PMC11578643; doi:10.1093/eurheartj/ehae557)
Supplement: ehae557_Supplementary_Data [file ehae557_supplementary_data.docx]

Table of Contents

[SUPPLEMENTARY METHODS 2](#_Toc161781270)

[Table S1: Definition of ASCVD 2](#_Toc161781271)

[Table S2: Exclusion criteria for CRP measurements and for patients 3](#_Toc161781272)

[Figure S1 Graphical depiction of the study design. 4](#_Toc161781273)

[Table S3: Algorithms to define comorbid conditions and ongoing medications 5](#_Toc161781274)

[Table S4: Algorithms to define study outcomes 6](#_Toc161781275)

[SUPPLEMENTARY RESULTS 7](#_Toc161781276)

[Figure S2. Number of eligible CRP measurements per participant during the baseline 3-month eligibility window (Panel A) and distribution of the geometric mean of these CRP values (Panel B), that defines the baseline CRP of the study 7](#_Toc161781277)

[Table S5: Baseline Characteristics of Adults with ASCVD, Overall and by four CRP categories 8](#_Toc161781278)

[Table S6: Univariable and multivariable-adjusted Logistic Regression Analysis of baseline conditions associated with CRP ≥2 mg/L. 10](#_Toc161781279)

[Figure S3. Cumulative incidence curves depicting the cumulative incidence of (Panel A) major adverse cardiovascular events (MACE), (Panel B) heart failure hospitalization and (Panel C) all-cause mortality; associated with four CRP categories in patients with atherosclerotic cardiovascular disease. 12](#_Toc161781280)

[Figure S4. Restricted cubic splines depicting the multivariable-adjusted hazard ratios (and 95% CIs) of (a) major adverse cardiovascular events (MACE); (b) heart failure hospitalization and (c) all-cause mortality associated with CRP (continuous, per mg/L higher). 13](#_Toc161781281)

[Figure S5. Subgroup analyses: Forest plots of CRP≥2 mg/L (versus CRP<2 mg/L) and rate of heart failure hospitalization 14](#_Toc161781282)

[Figure S6. Subgroup analyses: Forest plots of CRP≥2 mg/L (versus CRP<2 mg/L) and rate of all-cause mortality 15](#_Toc161781283)

[Table S7. Sensitivity analysis: Patient characteristics across CRP categories after redefining the baseline CRP with the minimum CRP encountered in the 3-month eligibility window. 16](#_Toc161781284)

[Table S8. Sensitivity analysis: number of events and hazard ratios for the risk of adverse outcomes associated with baseline CRP categories after exclusion of patients with a baseline CRP>10 mg/L. 18](#_Toc161781285)

[Table S9. Sensitivity analysis: number of events and hazard ratios for the risk of adverse outcomes associated with baseline CRP categories after exclusion of early events (within the first 6 or 12 months of follow-up). 18](#_Toc161781286)

# **SUPPLEMENTARY METHODS**

## **Table S1**: Definition of ASCVD

| Coronary event (Acute myocardial infarction, angina) | ICD-10 I20, I21, I22, I23, I24, I25 |
| --- | --- |
| Cerebrovascular event (stroke and transient ischemic attack) | ICD-10 I63, G459 |
| Peripheral vascular disease | ICD-10 I73.8, I73.9, I70 |
| Coronary revascularization | Procedure codes FNA-H, FNK, FNW |

International Classification of Diseases (ICD)-10th version (Sweden).

## **Table S2**: Exclusion criteria for CRP measurements and for patients

|  | **ICD-10 and/or ATC codes** |
| --- | --- |
| Exclusion criteria for CRP tests |  |
| CRP tests performed within 30 days after the (most recent) ASCVD diagnosis |  |
| CRP tests followed by the dispensation of any antibiotic/anti-mycotic or antiviral within 7 days. | J01 J02, J04, J05 |
| CRP tests during the following 3 months after dispensation of infectious medications | J01 J02, J04, J05 |
| CRP tests during in-hospital stays and in ER (±1 day from admission/discharge.) | Hospital measurements at admission of an elective surgery/procedure are accepted |
| A very high elevated CRP (>20 mg/L) |  |
| Exclusion criteria for patients with a first eligible CRP tests |  |
| Patients receiving corticosteroids in the 3 months before or after the first eligible CRP test | H02 |
| Patients with a diagnosis of chronic infections: tuberculosis, hepatitis, HIV at time of the first eligible CRP test | A15-A19, B15-B24 |
| Patients receiving immunosuppressants at time of the first eligible CRP test | L04 |

## **Figure S1** Graphical depiction of the study design.


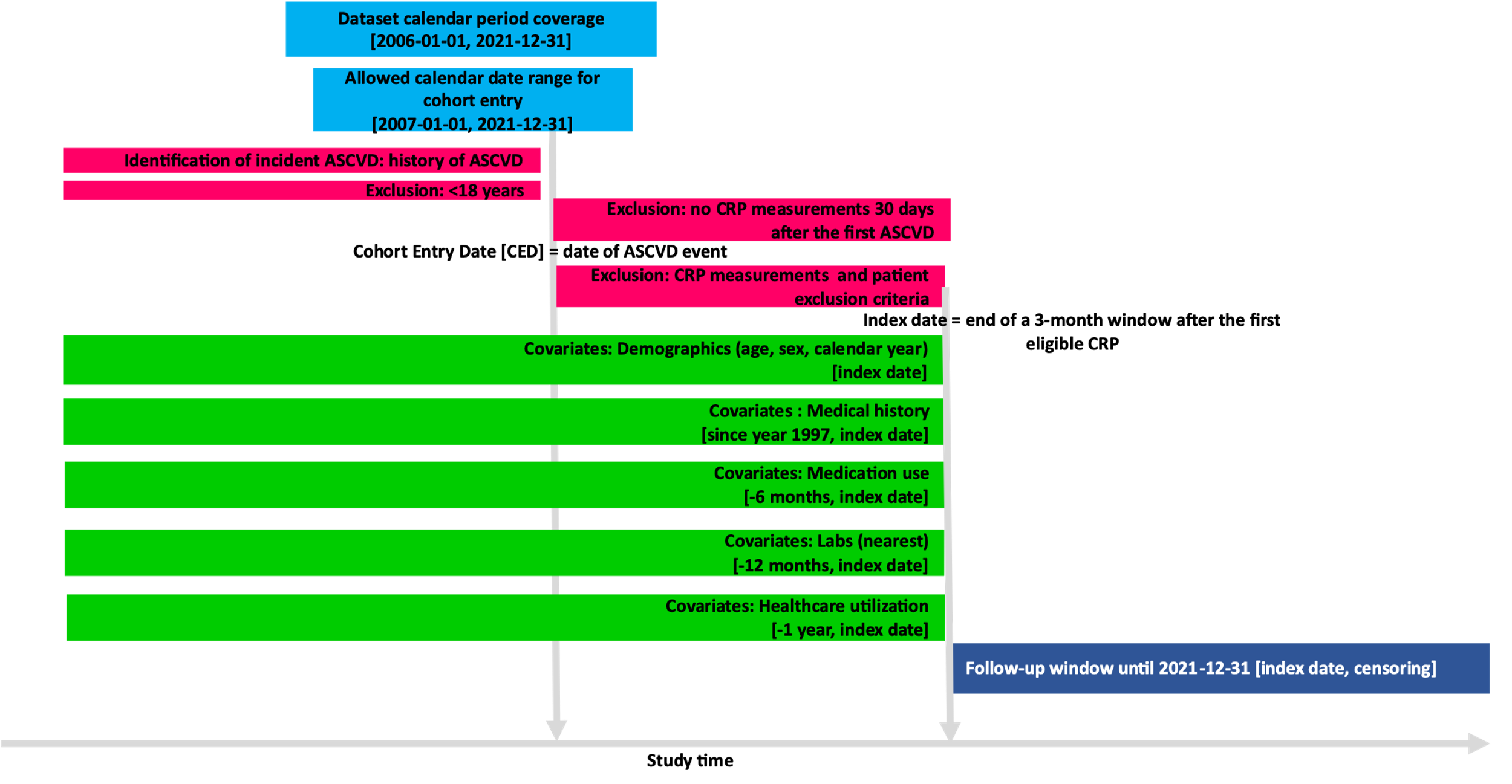


This graphical depiction of study design shows the different time windows used to define exposure, exclusion criteria, baseline covariates and outcomes.

Adapted from *Schneeweiss et al, Ann Int Med, 2019*.

## **Table S3**: Algorithms to define comorbid conditions and ongoing medications

| **Comorbid condition** | **ICD-10 or NOMESCO codes** | **ATC code** |
| --- | --- | --- |
| Chronic infections (tuberculosis, hepatitis, and HIV) | A15-A19, B15-B24 |  |
| Diabetes | E10 – E14 | A10 |
| Hypertension | I10 – I15 |  |
| Chronic obstructive pulmonary disease | J40 – J47 |  |
| Cancer (last three years) | Any code starting with C |  |
| Dementia | G30 – G31 |  |
| Myocardial infarction | I21, I22, I23, I24, I25 |  |
| Heart failure | I50, I110, I113, I971, K761, P290 |  |
| Peripheral vascular disease | I73.9, I70 |  |
| Angina | I20 |  |
| Stroke/TIA | I63-I69, G459 |  |
| Atrial fibrillation | I48 |  |
| Coronary artery bypass graft | FNA – FNW except FNG |  |
| Percutaneous coronary intervention | FNG00 – FNG06 |  |
| Inflammatory bowel diseases | K50 – K51 |  |
| Rheumatoid diseases | L40, L93, M05, M06, M10, M140, M315, M353, M32, M34 |  |
| **Medication** |  | **ATC code** |
| Corticosteroids |  | H02 |
| Immunosuppressant |  | L04 |
| Antibiotics, antivirals, antimycotics |  | J01, J02, J05, D06AA and D06AX |
| Aspirin |  | B01AC06, N02BA01 |
| Non-steroid anti-inflammatory |  | M01A |
| Ace inhibitors/Angiotensin |  | C09A – C09D |
| MRA |  | C03DA01 |
| Beta blocker |  | C07 |
| Diuretics |  | C03 |
| Calcium channel blockers |  | C08C – C08D |
| Digoxin |  | C01AA05 |
| Antidiabetics |  | A10 |
| Statins |  | C10AA, C10B |
| Ezetimibe |  | C10AX |
| Fibrates, resins, nicotinic acid |  | C10AB – C10AD |
| Other blood pressure medications |  | C02, C08E, C08G |

International Classification of Diseases (ICD)-10th version (Sweden), Nordic Medico-Statistical Committee (NOMESCO) classification of surgical procedures and Anatomical Therapeutic Chemical (ATC) codes (World Health Organization).

## **Table S4**: Algorithms to define study outcomes

| Outcome | ICD-10 Codes |
| --- | --- |
| Major adverse cardiac events (MACE) composite of: |  |
| Myocardial infarction | ICD-10 I21, I22, I23 |
| Ischemic stroke | I63 |
| All-cause mortality |  |
| Heart failure hospitalization | I110, I130, I132, I50 |
| CVD death | Death caused by  Myocardial infarction: I21, I22, I252  Congestive heart failure: I099, I110, I130, I132, I255, I420, I425-I429, I43, I50, P290  Cerebrovascular disease: I70, I71, I731, I738, I739, I771, I790, I792, K551, K558, K559, Z958, Z959  Peripheral vascular disease: G45-G46, H340, I60-I69  Atrial fibrillation: I48 |
| Non-CVD death | Any death not classified as CVD death |

# **SUPPLEMENTARY RESULTS**

## **Figure S2**. Number of eligible CRP measurements per participant during the baseline 3-month eligibility window (Panel A) and distribution of the geometric mean of these CRP values (Panel B), that defines the baseline CRP of the study


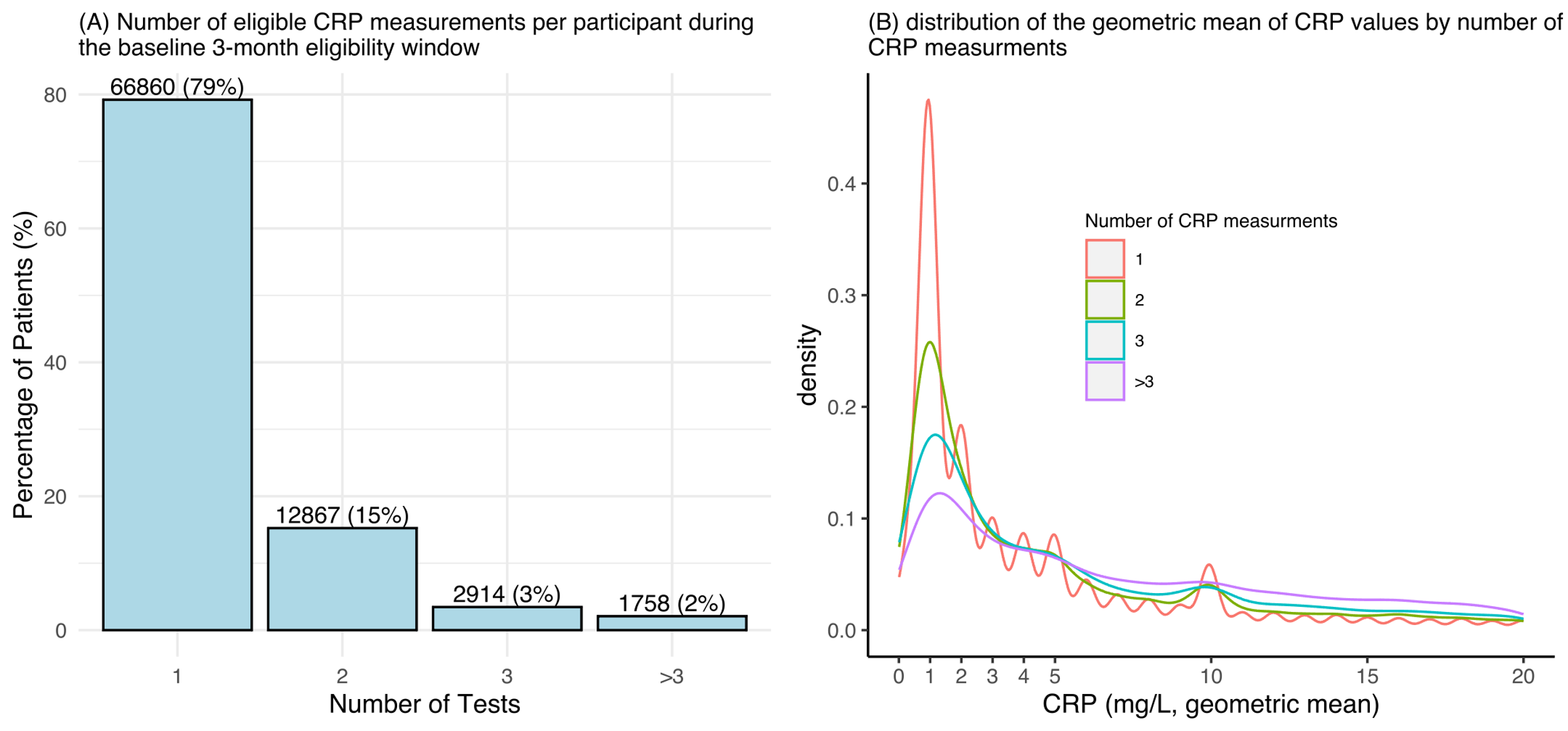


## **Table S5:** Baseline Characteristics of Adults with ASCVD, Overall and by four CRP categories

| **Characteristic** | **Overall**,  N = 84,399 | **CRP level** **<1 mg/L**,  N = 19,072 | **CRP level** **1-3 mg/L**,  N = 31,450 | **CRP level** **>3–10 mg/L**,  N = 25,770 | **CRP level** **>10-20 mg/L**,  N = 8,107 | **SMD** |
| --- | --- | --- | --- | --- | --- | --- |
| **CRP**; median [Q1-Q3] | 2.0 [1.0, 5.4] | 0.9 [0.7, 0.9] | 2.0 [1.0, 2.1] | 5.5 [4.1, 8.0] | 14.0 [12.0, 16.9] | 7.525 |
| **Age** in years; mean [SD] | 71 [13] | 68 [13] | 70 [13] | 72 [13] | 75 [13] | 0.530 |
| **Men** | 45,840 (54%) | 10,963 (57%) | 17,483 (56%) | 13,218 (51%) | 4,176 (52%) | 0.124 |
| **Time since ASCVD** |  |  |  |  |  |  |
| <6 months | 12,800 (15%) | 2,890 (15%) | 4,931 (16%) | 3,747 (15%) | 1,232 (15%) | 0.031 |
| ≥6 months - < 2 years | 41,407 (49%) | 9,745 (51%) | 15,417 (49%) | 12,492 (48%) | 3,753 (46%) | 0.096 |
| 2 years-<5 years | 20,861 (25%) | 4,575 (24%) | 7,454 (24%) | 6,651 (26%) | 2,181 (27%) | 0.073 |
| ≥ 5 years | 9,331 (11%) | 1,862 (9.8%) | 3,648 (12%) | 2,880 (11%) | 941 (12%) | 0.058 |
| **Hemoglobin,** g/dL; mean [SD] (n= 79,410) | 35 [16] | 138 [14] | 136 [15] | 133 [16] | 127 [17] | 0.667 |
| **LDL-c**, g/dL; mean [SD] (52,966) | 2.52 [1.01] | 2.45 [0.97] | 2.48 [1.01] | 2.62 [1.03] | 2.58 [1.03] | 0.165 |
| **eGFR categories** |  |  |  |  |  |  |
| ≥60 ml/min/1.73m^2^ | 66,380 (79%) | 16,283 (85%) | 25,189 (80%) | 19,270 (75%) | 5,638 (70%) | -0.170 |
| ≥30-≤59 ml/min/1.73m^2^ | 16,158 (19%) | 2,599 (14%) | 5,735 (18%) | 5,730 (22%) | 2,094 (26%) | 0.307 |
| ≤29 ml/min/1.73m^2^ | 1,861 (2.2%) | 190 (1.0%) | 526 (1.7%) | 770 (3.0%) | 375 (4.6%) | 0.230 |
| **Albuminuria categories** |  |  |  |  |  |  |
| <30 mg/g | 40,831 (48%) | 10,377 (54%) | 15,335 (49%) | 12,143 (47%) | 2,976 (37%) | 0.357 |
| 30-300 mg/g | 10,643 (13%) | 2,064 (11%) | 3,760 (12%) | 3,615 (14%) | 1,204 (15%) | 0.120 |
| >300 mg/g | 5,087 (6.0%) | 831 (4.4%) | 1,731 (5.5%) | 1,881 (7.3%) | 644 (7.9%) | 0.148 |
| missing | 27,838 (33%) | 5,800 (30%) | 10,624 (34%) | 8,131 (32%) | 3,283 (40%) | 0.213 |
| **Comorbid conditions** |  |  |  |  |  |  |
| Diabetes mellitus | 18,735 (22%) | 3,340 (18%) | 6,715 (21%) | 6,496 (25%) | 2,184 (27%) | 0.225 |
| Hypertension | 58,246 (69%) | 11,929 (63%) | 21,613 (69%) | 18,630 (72%) | 6,074 (75%) | 0.270 |
| Myocardial infraction | 31,442 (37%) | 7,677 (40%) | 12,104 (38%) | 8,945 (35%) | 2,716 (34%) | 0.140 |
| CABG | 3,137 (3.7%) | 639 (3.4%) | 1,226 (3.9%) | 975 (3.8%) | 297 (3.7%) | 0.029 |
| PCI | 15,171 (18%) | 4,368 (23%) | 6,243 (20%) | 3,685 (14%) | 875 (11%) | 0.325 |
| Angina | 24,998 (30%) | 6,259 (33%) | 9,448 (30%) | 7,360 (29%) | 1,931 (24%) | 0.199 |
| Heart failure | 14,646 (17%) | 2,174 (11%) | 4,789 (15%) | 5,288 (21%) | 2,395 (30%) | 0.467 |
| Stroke/TIA | 36,088 (43%) | 7,722 (40%) | 13,187 (42%) | 11,296 (44%) | 3,883 (48%) | 0.149 |
| Peripheral vascular disease | 9,427 (11%) | 1,512 (7.9%) | 3,190 (10%) | 3,378 (13%) | 1,347 (17%) | 0.269 |
| Atrial fibrillation | 17,005 (20%) | 2,791 (15%) | 5,846 (19%) | 5,940 (23%) | 2,428 (30%) | 0.376 |
| Rheumatoid disease | 9,776 (12%) | 1,459 (7.6%) | 3,406 (11%) | 3,617 (14%) | 1,294 (16%) | 0.255 |
| Chronic respiratory disease | 15,356 (18%) | 2,602 (14%) | 5,485 (17%) | 5,438 (21%) | 1,831 (23%) | 0.230 |
| Recent Cancer (3-years) | 10,722 (13%) | 1,966 (10%) | 3,710 (12%) | 3,711 (14%) | 1,335 (16%) | 0.182 |
| Recent Anemia (1-year) | 27,988 (33%) | 4,657 (24%) | 9,454 (30%) | 9,761 (38%) | 4,116 (51%) | 0.561 |
| Dyslipidemia | 60,951 (72%) | 14,936 (78%) | 23,762 (76%) | 17,445 (68%) | 4,808 (59%) | 0.421 |
| **Ongoing medications** |  |  |  |  |  |  |
| Antiplatelets | 54,998 (65%) | 13,428 (70%) | 20,927 (67%) | 15,808 (61%) | 4,835 (60%) | 0.225 |
| ACEIs/ARBs | 45,730 (54%) | 10,078 (53%) | 17,599 (56%) | 13,919 (54%) | 4,134 (51%) | 0.099 |
| MRAs | 4,873 (5.8%) | 735 (3.9%) | 1,662 (5.3%) | 1,741 (6.8%) | 735 (9.1%) | 0.216 |
| β-Blockers | 44,017 (52%) | 9,882 (52%) | 16,395 (52%) | 13,489 (52%) | 4,251 (52%) | 0.012 |
| SGLT-2 inhibitors | 705 (0.8%) | 90 (0.5%) | 342 (1.1%) | 226 (0.9%) | 47 (0.6%) | 0.071 |
| Diuretics | 21,020 (25%) | 3,140 (16%) | 6,901 (22%) | 7,892 (31%) | 3,087 (38%) | 0.496 |
| Calcium channel blockers | 22,681 (27%) | 4,480 (23%) | 8,791 (28%) | 7,288 (28%) | 2,122 (26%) | 0.108 |
| Statins/PCSK-9i, Ezetimibe | 49,809 (59%) | 12,592 (66%) | 19,662 (63%) | 13,836 (54%) | 3,719 (46%) | 0.412 |
| NSAIDs | 10,328 (12%) | 2,079 (11%) | 3,803 (12%) | 3,548 (14%) | 898 (11%) | 0.088 |
|  | | | | | |  |

## **Table S6**: Univariable and multivariable-adjusted Logistic Regression Analysis of baseline conditions associated with CRP ≥2 mg/L.

|  | **Univariable** | | **Multivariable** | |  |
| --- | --- | --- | --- | --- | --- |
|  | **OR (95% CI)** | **p-value** | **OR (95% CI)** | **p-value** |  |
|  |  |  |  |  |  |
| **Age,** per 5 years increment | 1.10 (1.09-1.10) | <0.001 | 1.04 (1.03-1.04) | <0.0001 |  |
| **Sex** |  |  |  |  |  |
| Women | REF | - | REF | - |  |
| Men | 0.82 (0.80-0.85) | <0.0001 | 0.96 (0.93-0.99) | 0.004 |  |
| **Time since ASCVD** |  |  |  |  |  |
| <6 months | REF | - | REF | - |  |
| ≥6 months - < 2 years | 0.99 (0.95-1.03) | 0.622 | 0.89 (0.86-0.93) | <0.0001 |  |
| 2 years-<5 years | 1.11 (1.06-1.16) | <0.0001 | 0.97 (0.93-1.02) | 0.32 |  |
| ≥5 years | 1.06 (1.00-1.12) | 0.035 | 0.92 (0.87-0.97) | 0.011 |  |
| **eGFR categories** (KDIGO G categories) |  |  |  |  |  |
| ≥60 ml/min/1.73m2 | REF | - | REF | - |  |
| ≥30-≤59 ml/min/1.73m2 | 1.69 (1.62-1.75) | <0.001 | 1.15 (1.10-1.20) | <0.001 |  |
| ≤29 ml/min/1.73m2 | 2.72 (2.43-3.03) | <0.001 | 1.24 (1.10-1.40) | <0.001 |  |
| **Albuminuria (KDIGO A stages)** |  |  |  |  |  |
| A1 (ACR < 30 mg/g) | REF | - | REF | - |  |
| A2 (ACR 30-300 mg/g) | 1.45 (1.39-1.52) | <0.0001 | 1.20 (1.14-1.26) | <0.001 |  |
| A3 (ACR > 300 mg/g) | 1.79 (1.65-1.94) | <0.001 | 1.27 (1.16-1.38) | <0.001 |  |
| missing | 1.12 (1.09-1.15) | <0.001 | 1.02 (0.99-1.05) | 0.221 |  |
| **Comorbid conditions** |  |  |  |  |  |
| Diabetes Mellitus | 1.40 (1.36-1.45) | <0.0001 | 1.30 (1.24-1.36) | <0.0001 |  |
| Hypertension | 1.43 (1.39-1.47) | <0.0001 | 1.24 (1.20-1.29) | <0.0001 |  |
| Myocardial infraction | 0.82 (0.80-0.84) | <0.0001 | 1.04 (0.99-1.09) | 0.125 |  |
| CABG | 1.09 (1.01-1.17) | 0.021 | 1.25 (1.15-1.36) | <0.0001 |  |
| PCI | 0.60 (0.58-0.62) | <0.0001 | 0.81 (0.77-0.85) | <0.0001 |  |
| Angina | 0.86 (0.84-0.89) | <0.001 | 0.88 (0.85-0.91) | <0.0001 |  |
| Heart failure | 1.84 (1.77-1.91) | <0.0001 | 1.09 (1.02-1.16) | 0.014 |  |
| Stroke | 1.12 (1.08-1.15) | <0.0001 | 1.10 (1.05-1.15) | <0.001 |  |
| Peripheral vascular disease | 1.61 (1.54-1.69) | <0.0001 | 1.38 (1.30-1.46) | <0.001 |  |
| Atrial fibrillation | 1.58 (1.53-1.64) | <0.0001 | 1.13 (1.09-1.18) | <0.0001 |  |
| Rheumatoid disease | 1.65 (1.58-1.73) | <0.0001 | 1.38 (1.32-1.45) | <0.0001 |  |
| Chronic respiratory disease | 1.44 (1.39-1.50) | <0.0001 | 1.29 (1.24-1.34) | <0.0001 |  |
| Recent Cancer (3-years) | 1.38 (1.32-1.44) | <0.0001 | 1.20 (1.15-1.26) | <0.0001 |  |
| Recent Anemia (1-year) | 1.70 (1.65-1.75) | <0.0001 | 1.31 (1.27-1.35) | <0.0001 |  |
| **Ongoing medications** |  |  |  |  |  |
| Antiplatelets | 0.72 (0.70-0.75) | <0.0001 | 0.93 (0.90-0.96) | <0.0001 |  |
| ACEIs/ARBs | 0.99 (0.96-1.02) | 0.656 | 0.90 (0.87-0.93) | <0.0001 |  |
| MRA | 1.56 (1.47-1.66) | <0.0001 | 0.89 (0.82-0.95) | 0.001 |  |
| β-Blockers | 1.04 (1.01-1.07) | 0.005 | 1.01 (0.98-1.05) | 0.473 |  |
| SGLT-2 inhibitors | 0.90 (0.78-1.03) | 0.508 | 0.88 (0.75-1.04) | 0.131 |  |
| Diuretics | 1.97 (1.90-2.04) | <0.0001 | 1.43 (1.37-1.49) | <0.0001 |  |
| Calcium channel blockers | 1.16 (1.12-1.19) | <0.0001 | 1.05 (1.01-1.09) | 0.007 |  |
| Statins/PCSK-9i, Ezetimibe | 0.60 (0.58-0.61) | <0.0001 | 0.65 (0.62-0.67) | <0.0001 |  |
| NSAIDs | 1.20 (1.15-1.25) | <0.0001 | 1.28 (1.23-1.34) | <0.0001 |  |

## **Figure S3**. Cumulative incidence curves depicting the cumulative incidence of (Panel A) major adverse cardiovascular events (MACE), (Panel B) heart failure hospitalization and (Panel C) all-cause mortality; associated with four CRP categories in patients with atherosclerotic cardiovascular disease.


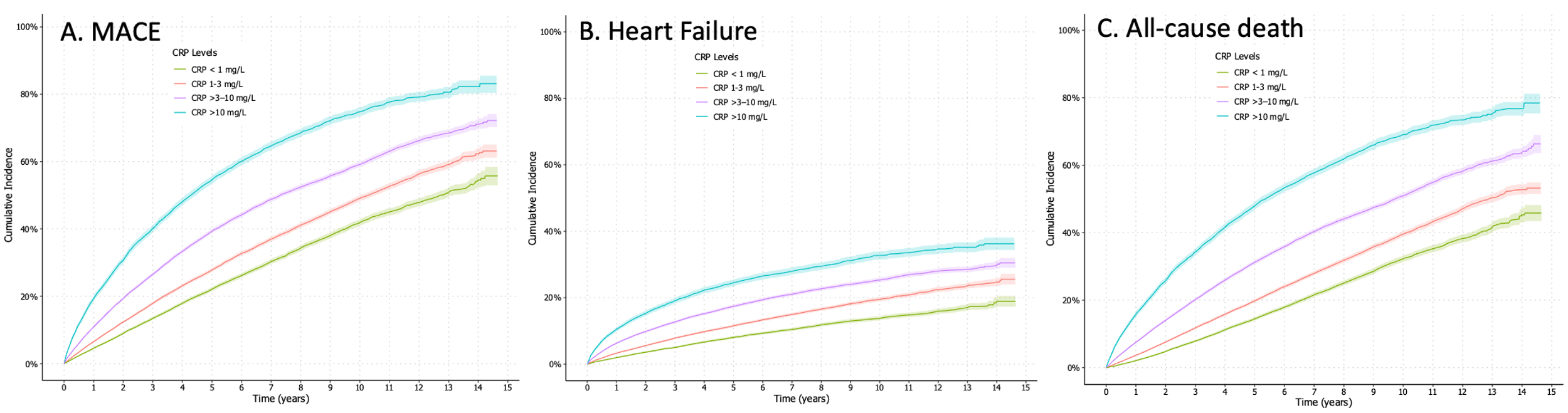


## **Figure S****4**. Restricted cubic splines depicting the multivariable-adjusted hazard ratios (and 95% CIs) of (a) major adverse cardiovascular events (MACE); (b) heart failure hospitalization and (c) all-cause mortality associated with CRP (continuous, per mg/L higher).


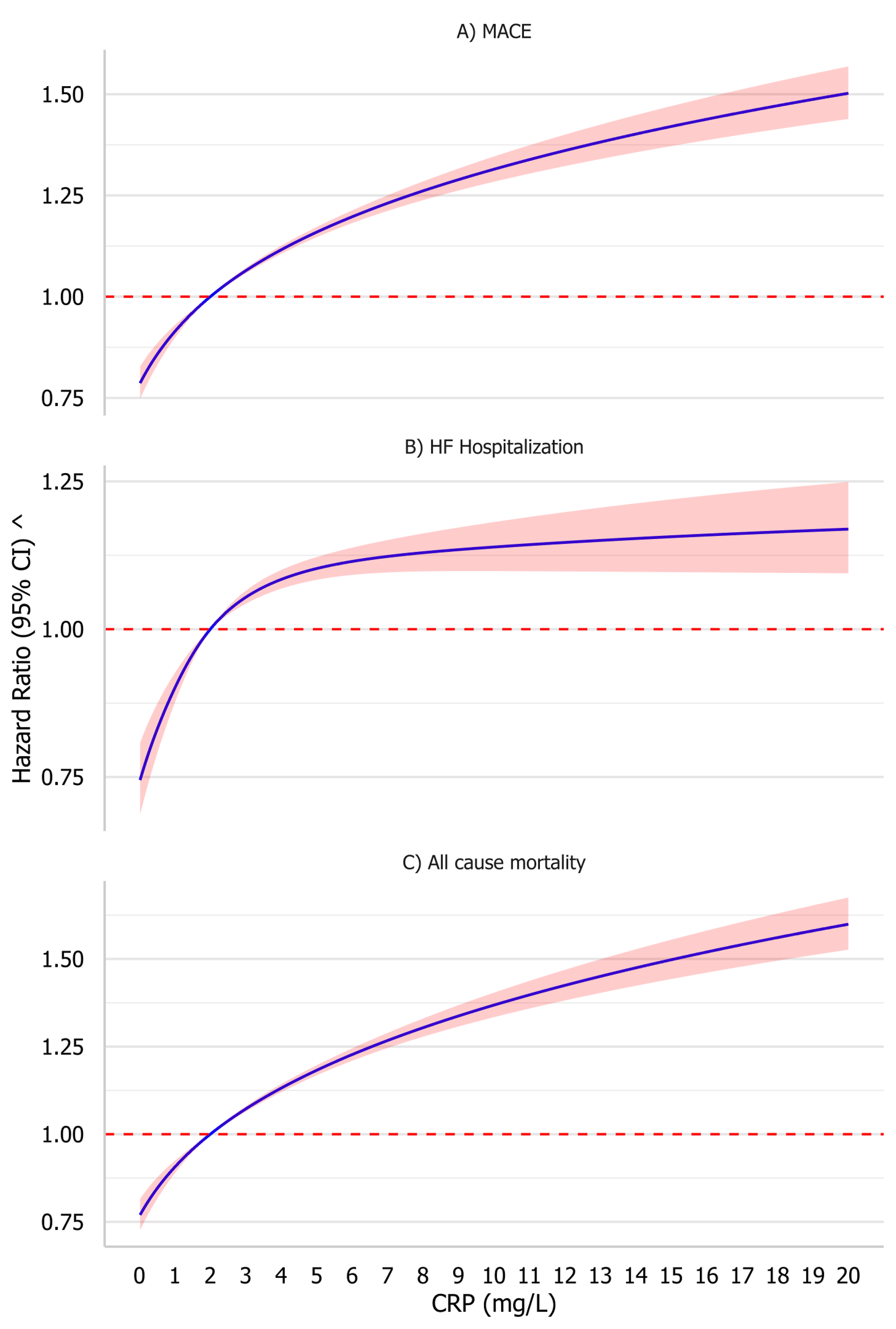


^ adjusted for age, sex, time since ASCVD, eGFR, albuminuria, comorbidities (diabetes mellitus, hypertension, chronic respiratory disease, cancer, MI, angina, heart failure, peripheral vascular disease, stroke/TIA, atrial fibrillation, and rheumatoid diseases), undertaken procedures (coronary artery bypass grafting and percutaneous coronary intervention), and ongoing medications (antiplatelet, NSAIDs, angiotensin‐converting enzyme inhibitors/angiotensin receptor blockers, mineralocorticoid‐receptor antagonists, β blocker, SGLT-2i, diuretics, calcium channel blockers, digoxin, lipid-lowering treatment [statins, PCSk9i, ezetimibe]).

## **Figure S5**. Subgroup analyses: Forest plots of CRP≥2 mg/L (versus CRP<2 mg/L) and rate of heart failure hospitalization

**
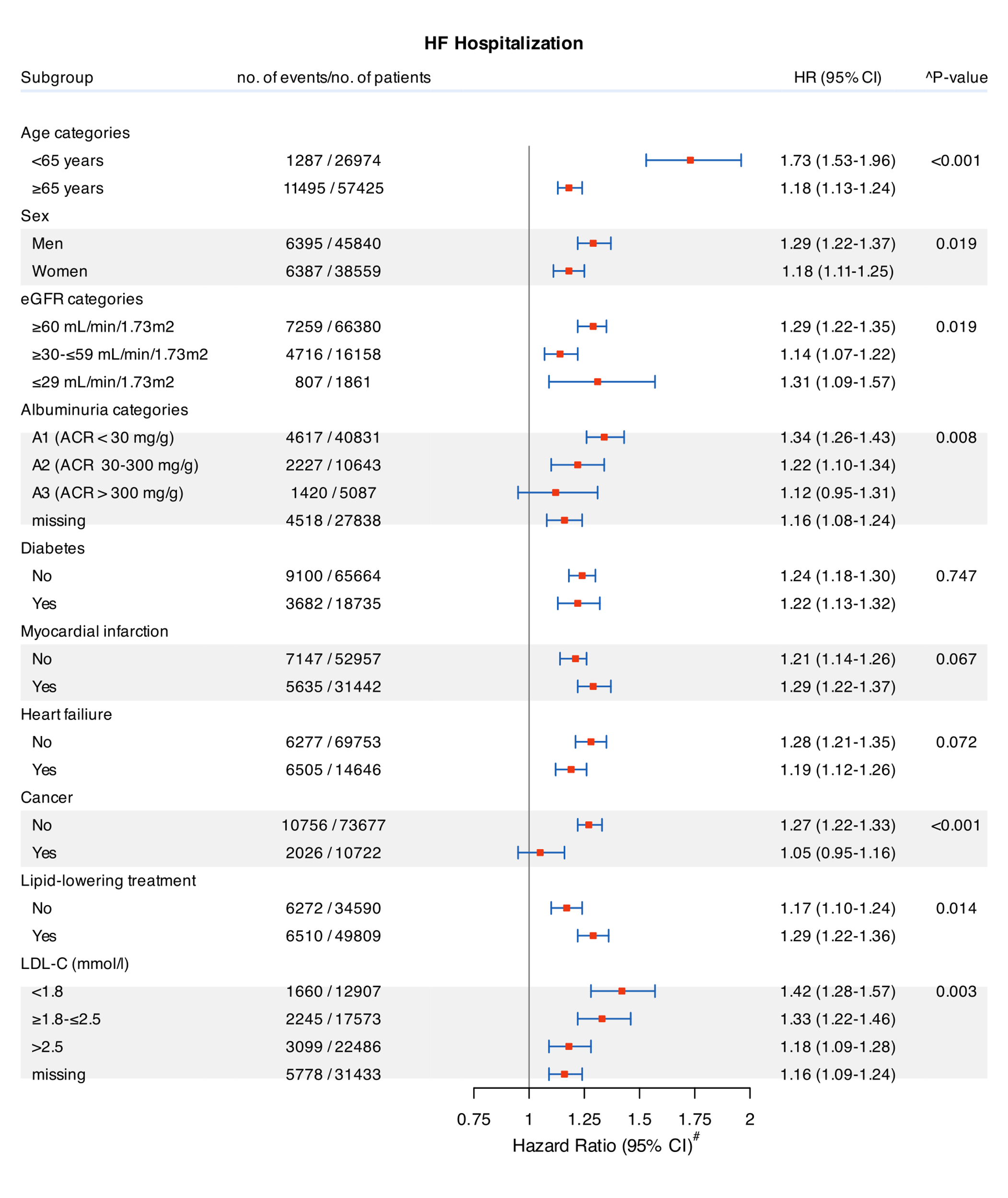
**

^ Models were adjusted (when appropriate) for age, sex, time since ASCVD, eGFR, albuminuria, comorbidities (diabetes mellitus, hypertension, chronic respiratory disease, cancer, MI, angina, heart failure, peripheral vascular disease, stroke/TIA, atrial fibrillation, and rheumatoid diseases), undertaken procedures (coronary artery bypass grafting and percutaneous coronary intervention), and ongoing medications (antiplatelet, NSAIDs, angiotensin‐converting enzyme inhibitors/angiotensin receptor blockers, mineralocorticoid‐receptor antagonists, β blocker, SGLT-2i, diuretics, calcium channel blockers, digoxin, lipid-lowering treatment [statins, PCSk9i, ezetimibe]).

## **Figure S6**. Subgroup analyses: Forest plots of CRP≥2 mg/L (versus CRP<2 mg/L) and rate of all-cause mortality


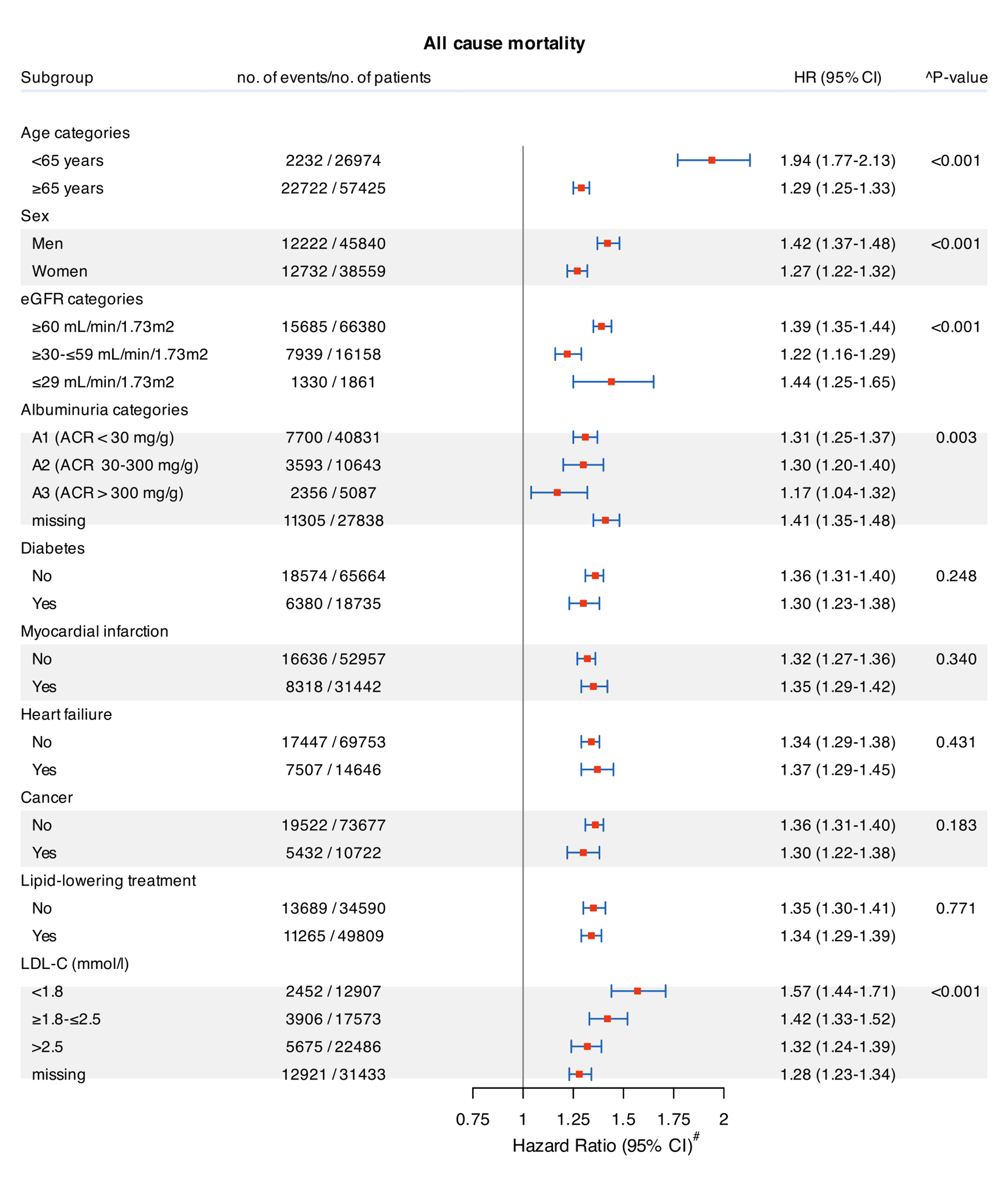


^ Models were adjusted (when appropriate) for age, sex, time since ASCVD, eGFR, albuminuria, comorbidities (diabetes mellitus, hypertension, chronic respiratory disease, cancer, MI, angina, heart failure, peripheral vascular disease, stroke/TIA, atrial fibrillation, and rheumatoid diseases), undertaken procedures (coronary artery bypass grafting and percutaneous coronary intervention), and ongoing medications (antiplatelet, NSAIDs, angiotensin‐converting enzyme inhibitors/angiotensin receptor blockers, mineralocorticoid‐receptor antagonists, β blocker, SGLT-2i, diuretics, calcium channel blockers, digoxin, lipid-lowering treatment [statins, PCSk9i, ezetimibe]).

## **Table S7**. Sensitivity analysis: Patient characteristics across CRP categories after redefining the baseline CRP with the minimum CRP encountered in the 3-month eligibility window.

| **Characteristic** | **Overall**,  N = 84,399 | **CRP Level <2 mg/L**,  N = 36,890 (40%) | **CRP Level ≥2-20 mg/L**,  N = 47,509 (59%) | **SMD** |
| --- | --- | --- | --- | --- |
| **CRP** in mg/L; median [Q1-Q3] | 2.0 [1.0, 5.4] | 0.9 [0.9, 1.0] | 4.9 [3.0, 9.0] | 1.7 |
| **Age** in years; mean [SD] | 71 [13] | 69 [13] | 72 [13] | 0.24 |
| **Men** | 45,840 (54%) | 19,742 (57%) | 26,098 (52%) | -0.10 |
| **Time since ASCVD** |  |  |  |  |
| <6 months | 12,800 (15%) | 5,828 (16%) | 6,972 (15%) | -0.03 |
| ≥6 months - <2 years | 41,407 (49%) | 18,504 (50%) | 22,903 (48%) | -0.03 |
| 2 years-<5 years | 20,861 (25%) | 8,620 (23%) | 12,241 (26%) | 0.05 |
| ≥5 years | 9,331 (11%) | 3,938 (11%) | 5,393 (11%) | 0.02 |
| **Hemoglobin** in g/dL (n=79,410) | 135 [16] | 137 [15] | 133 [17] | -0.23 |
| **LDL-c** in g/dL (n=52,966) | 2.52 [1.01] | 2.42 [0.98] | 2.61 [1.03] | 0.18 |
| **eGFR categories** |  |  |  |  |
| ≥60 ml/min/1.73m^2^ | 66,380 (79%) | 30,806 (84%) | 35,574 (75%) | -0.22 |
| ≥30-≤59 ml/min/1.73m^2^ | 16,158 (19%) | 5,601 (15%) | 10,557 (22%) | 0.18 |
| ≤30 ml/min/1.73m^2^ | 1,861 (2.2%) | 483 (1.3%) | 1,378 (2.9%) | 0.11 |
| **Albuminuria categories** |  |  |  |  |
| <30 mg/g | 40,831 (48%) | 19,082 (52%) | 21,749 (46%) | -0.11 |
| 30-300 mg/g | 10,643 (13%) | 4,015 (11%) | 6,628 (14%) | 0.09 |
| >300 mg/g | 5,087 (6.0%) | 1,776 (4.8%) | 3,311 (7.0%) | 0.09 |
| missing | 27,838 (33%) | 12,017 (33%) | 15,821 (33%) | 0.01 |
| **Comorbid conditions** |  |  |  |  |
| Diabetes mellitus | 18,735 (22%) | 7,031 (19%) | 11,704 (25%) | 0.135 |
| Hypertension | 58,246 (69%) | 23,918 (65%) | 34,328 (72%) | 0.16 |
| Myocardial infraction | 31,442 (37%) | 14,765 (40%) | 16,677 (35%) | -0.10 |
| CABG | 3,137 (3.7%) | 1,372 (3.7%) | 1,765 (3.7%) | -0.00 |
| PCI | 15,171 (18%) | 8,193 (22%) | 6,978 (15%) | -0.19 |
| Angina | 24,998 (30%) | 11,579 (31%) | 13,419 (28%) | -0.06 |
| Heart failure | 14,646 (17%) | 4,754 (13%) | 9,892 (21%) | 0.21 |
| Stroke/TIA | 36,088 (43%) | 15,299 (41%) | 20,789 (44%) | 0.04 |
| Peripheral vascular disease | 9,427 (11%) | 3,178 (8.6%) | 6,249 (13%) | 0.14 |
| Atrial fibrillation | 17,005 (20%) | 6,012 (16%) | 10,993 (23%) | 0.17 |
| Rheumatoid disease | 9,776 (12%) | 3,276 (8.9%) | 6,500 (14%) | 0.15 |
| Chronic respiratory disease | 15,356 (18%) | 5,679 (15%) | 9,677 (20%) | 0.13 |
| Recent Cancer (3-years) | 10,722 (13%) | 4,054 (11%) | 6,668 (14%) | 0.09 |
| Recent Anemia (1-year) | 27,988 (33%) | 10,369 (28%) | 17,619 (37%) | 0.19 |
| Dyslipidemia | 60,951 (72%) | 27,069 (78%) | 33,882 (68%) | -0.23 |
| **Ongoing medications** |  |  |  |  |
| Antiplatelets | 54,998 (65%) | 25,520 (69%) | 29,478 (62%) | -0.15 |
| ACEIs/ARBs | 45,730 (54%) | 20,012 (54%) | 25,718 (54%) | -0.00 |
| MRAs | 4,873 (5.8%) | 1,683 (4.6%) | 3,190 (6.7%) | 0.09 |
| β-Blockers | 44,017 (52%) | 19,106 (52%) | 24,911 (52%) | 0.01 |
| SGLT-2 inhibitors | 705 (0.8%) | 326 (0.9%) | 379 (0.8%) | -0.00 |
| Diuretics | 21,020 (25%) | 6,760 (18%) | 14,260 (30%) | 0.27 |
| Calcium channel blockers | 22,681 (27%) | 9,318 (25%) | 13,363 (28%) | 0.06 |
| Statins/PCSK-9i, Ezetimibe | 49,809 (59%) | 24,301 (66%) | 25,508 (54%) | -0.25 |
| NSAIDs | 10,328 (12%) | 4,144 (11%) | 6,184 (13%) | 0.05 |

## **Table S8.** Sensitivity analysis: number of events and hazard ratios for the risk of adverse outcomes associated with baseline CRP categories after exclusion of patients with a baseline CRP>10 mg/L.

|  |  | **After excluding 8107 patients with CRP>10 mg/L** | |
| --- | --- | --- | --- |
|  | **Original analysis**  **(for comparison)**  **^Adj. HR (95% CI)** | **Number of events/**  **number of patients** | **^Adj. HR (95% CI)** |
| **MACE** |  |  |  |
| CRP<2 mg/L | REF. | 9331/34526 | REF. |
| CRP≥2 mg/L | 1.3 (1.27-1.33) | 16191/40756 | 1.24 (1.21-1.27) |
| **Cardiovascular mortality** |  |  |  |
| CRP<2 mg/L | REF. | 1736/34526 | REF. |
| CRP≥2 mg/L | 1.29 (1.22-1.36) | 3631/40756 | 1.22 (1.15-1.3) |
| **Non-cardiovascular mortality** |  |  |  |
| CRP<2 mg/L | REF. | 5176/34526 | REF. |
| CRP≥2 mg/L | 1.24 (1.2-1.28) | 9634/40756 | 1.19 (1.15-1.23) |
| **All-cause mortality** |  |  |  |
| CRP<2 mg/L | REF. | 6912/34526 | REF. |
| CRP≥2 mg/L | 1.35 (1.31-1.39) | 13265/40756 | 1.27 (1.23-1.31) |
| **Heart failure** |  |  |  |
| CRP<2 mg/L | REF. | 3409/34526 | REF. |
| CRP≥2 mg/L | 1.24 (1.2-1.3) | 7019/40756 | 1.22 (1.17-1.27) |

^ adjusted for age, sex, time since ASCVD, eGFR, albuminuria, comorbidities (diabetes mellitus, hypertension, chronic respiratory disease, cancer, MI, angina, heart failure, peripheral vascular disease, stroke/TIA, atrial fibrillation, and rheumatoid diseases), undertaken procedures (coronary artery bypass grafting and percutaneous coronary intervention), and ongoing medications (antiplatelet, NSAIDs, angiotensin‐converting enzyme inhibitors/angiotensin receptor blockers, mineralocorticoid‐receptor antagonists, β blocker, SGLT-2i, diuretics, calcium channel blockers, digoxin, lipid-lowering treatment [statins, PCSk9i, ezetimibe]).

## **Table S9.** Sensitivity analysis: number of events and hazard ratios for the risk of adverse outcomes associated with baseline CRP categories after exclusion of early events (within the first 6 or 12 months of follow-up).

|  |  | **After excluding events within 6 months** | | **After excluding events within 12 months** | |
| --- | --- | --- | --- | --- | --- |
|  | **Original analysis**  **(for comparison)**  **^Adj. HR (95% CI)** | **Number of events/**  **number of patients** | **^Adj. HR (95% CI)** | **Number of events/**  **number of patients** | **^Adj. HR (95% CI)** |
| **MACE** |  |  |  |  |  |
| CRP<2 mg/L | REF. | 8459/33654 | REF. | 7674/32869 | REF. |
| CRP≥2 mg/L | 1.3 (1.27-1.33) | 18425/46828 | 1.27 (1.24-1.30) | 16129/44532 | 1.24 (1.21-1.27) |
|  |  |  |  |  |  |
| CRP≤1 mg/L | REF. | 5215/18628 | REF. | 4801/18214 | REF. |
| CRP >1-3 mg/L | 1.09 (1.06-1.13) | 8190/30420 | 1.09 (1.05-1.12) | 7295/29525 | 1.08 (1.04-1.12) |
| CRP >3-10 mg/L | 1.34 (1.3-1.38) | 9590/24255 | 1.31 (1.27-1.36) | 8416/23081 | 1.29 (1.25-1.34) |
| CRP >10-20 mg/L | 1.61 (1.54-1.67) | 3889/7179 | 1.53 (1.46-1.59) | 3291/6581 | 1.49 (1.42-1.56) |
|  |  |  |  |  |  |
| **Cardiovascular mortality** |  |  |  |  |  |
| CRP<2 mg/L | REF. | 1631/34421 | REF. | 1519/34309 | REF. |
| CRP≥2 mg/L | 1.29 (1.22-1.36) | 4456/49266 | 1.24 (1.17-1.31) | 3955/48765 | 1.22 (1.15-1.3) |
|  |  |  |  |  |  |
| CRP≤1 mg/L | REF. | 950/19020 | REF. | 892/18962 | REF. |
| CRP >1-3 mg/L | 1.16 (1.07-1.25) | 1737/31302 | 1.16 (1.07-1.25) | 1584/31149 | 1.15 (1.06-1.25) |
| CRP >3-10 mg/L | 1.36 (1.26-1.47) | 2308/25480 | 1.32 (1.22-1.42) | 2057/25229 | 1.29 (1.19-1.39) |
| CRP >10-20 mg/L | 1.57 (1.44-1.71) | 1092/7885 | 1.46 (1.33-1.6) | 941/7734 | 1.40 (1.27-1.54) |
|  |  |  |  |  |  |
| **Non-cardiovascular mortality** |  |  |  |  |  |
| CRP<2 mg/L | REF. | 4887/34237 | REF. | 4589/33939 | REF. |
| CRP≥2 mg/L | 1.24 (1.2-1.28) | 11504/48398 | 1.2 (1.15-1.24) | 10316/47210 | 1.16 (1.12-1.2) |
|  |  |  |  |  |  |
| CRP≤1 mg/L | REF. | 3038/18942 | REF. | 2882/18786 | REF. |
| CRP >1-3 mg/L | 1.04 (1-1.09) | 4820/31063 | 1.04 (0.99-1.08) | 4436/30679 | 1.03 (0.98-1.08) |
| CRP >3-10 mg/L | 1.25 (1.19-1.3) | 6025/25055 | 1.2 (1.15-1.26) | 5422/24452 | 1.17 (1.12-1.23) |
| CRP >10-20 mg/L | 1.36 (1.29-1.43) | 2508/7575 | 1.24 (1.18-1.31) | 2165/7232 | 1.18 (1.12-1.25) |
|  |  |  |  |  |  |
| **All-cause mortality** |  |  |  |  |  |
| CRP<2 mg/L | REF. | 6518/34132 | REF. | 6108/33722 | REF. |
| CRP≥2 mg/L | 1.35 (1.31-1.39) | 15960/47791 | 1.3 (1.27-1.34) | 14271/46102 | 1.27 (1.23-1.31) |
|  |  |  |  |  |  |
| CRP≤1 mg/L | REF. | 3988/18890 | REF. | 3774/18676 | REF. |
| CRP >1-3 mg/L | 1.1 (1.06-1.14) | 6557/30915 | 1.09 (1.04-1.13) | 6020/30378 | 1.07 (1.03-1.12) |
| CRP >3-10 mg/L | 1.39 (1.34-1.44) | 8333/24765 | 1.35 (1.3-1.4) | 7479/23911 | 1.32 (1.27-1.38) |
| CRP >10-20 mg/L | 1.68 (1.61-1.75) | 3600/7353 | 1.57 (1.5-1.64) | 3106/6859 | 1.51 (1.44-1.59) |
|  |  |  |  |  |  |
| **Heart failure** |  |  |  |  |  |
| CRP<2 mg/L | REF. | 2993/34110 | REF. | 2667/33784 | REF. |
| CRP≥2 mg/L | 1.24 (1.2-1.3) | 7476/47976 | 1.18 (1.13-1.24) | 6343/46843 | 1.22 (1.17-1.27) |
|  |  |  |  |  |  |
| CRP≤1 mg/L | REF. | 1749/18860 | REF. | 1589/18700 | REF. |
| CRP >1-3 mg/L | 1.13 (1.07-1.2) | 3219/30877 | 1.14 (1.07-1.21) | 2804/30462 | 1.13 (1.06-1.21) |
| CRP >3-10 mg/L | 1.27 (1.2-1.34) | 3921/24799 | 1.22 (1.15-1.30) | 3312/24190 | 1.19 (1.12-1.27) |
| CRP >10-20 mg/L | 1.31 (1.22-1.39) | 1580/7550 | 1.21 (1.12-1.30) | 1305/7275 | 1.17 (1.09-1.27) |

^ adjusted for age, sex, time since ASCVD, eGFR, albuminuria, comorbidities (diabetes mellitus, hypertension, chronic respiratory disease, cancer, MI, angina, heart failure, peripheral vascular disease, stroke/TIA, atrial fibrillation, and rheumatoid diseases), undertaken procedures (coronary artery bypass grafting and percutaneous coronary intervention), and ongoing medications (antiplatelet, NSAIDs, angiotensin‐converting enzyme inhibitors/angiotensin receptor blockers, mineralocorticoid‐receptor antagonists, β blocker, SGLT-2i, diuretics, calcium channel blockers, digoxin, lipid-lowering treatment [statins, PCSk9i, ezetimibe]).
